# Improving estimates of the burden of severe wasting: analysis of secondary prevalence and incidence data from 352 sites

**DOI:** 10.1136/bmjgh-2020-004342

**Published:** 2021-03-02

**Authors:** Sheila Isanaka, Christopher T Andersen, Simon Cousens, Mark Myatt, André Briend, Julia Krasevec, Chika Hayashi, Amy Mayberry, Louise Mwirigi, Saul Guerrero

**Affiliations:** 1 Department of Nutrition, Harvard T.H. Chan School of Public Health, Boston, Massachusetts, USA; 2 Department of Research, Epicentre, Paris, France; 3 Department of Epidemiology, Harvard T.H. Chan School of Public Health, Boston, Massachusetts, USA; 4 London School of Hygiene and Tropical Medicine, London, UK; 5 Brixton Health, Llwyngwril, UK; 6 Center for Child Health Research, Faculty of Medicine and Medical Technology, Tampere University, Tampere, Finland; 7 Department of Nutrition, Exercise and Sports, Faculty of Science, University of Copenhagen, Frederiksberg, Denmark; 8 UNICEF, New York City, New York, USA; 9 No Wasted Lives & Action Against Hunger UK, London, UK

**Keywords:** child health, epidemiology, nutrition

## Abstract

**Introduction:**

Estimates of incident cases of severe wasting among young children are not available for most settings but are needed for optimal planning of treatment programmes and burden estimation. To improve programme planning, global guidance recommends a single ‘incidence correction factor’ of 1.6 be applied to available prevalence estimates to account for incident cases. This study aimed to update estimates of the incidence correction factor to improve programme planning and inform the approach to burden estimation for severe wasting.

**Methods:**

A global call was issued for secondary data from severe wasting treatment programmes including prevalence, population size, programme admission and programme coverage through a UNICEF-led effort. Site-specific incidence correction factors were calculated as the number of incident cases (annual programme admissions/programme coverage) divided by the number of prevalent cases (prevalence*population size). Estimates were aggregated by country, region and overall using inverse-variance weighted random-effects meta-analysis.

**Results:**

We estimated incidence correction factors from 352 sites in 20 countries. Estimates aggregated by country ranged from 1.3 (Nigeria) to 30.1 (Burundi). Excluding implausible values, the overall incidence correction factor was 3.6 (95% CI 3.4 to 3.9).

**Conclusion:**

Our results suggest that incidence correction factors vary between sites and that the burden of severe wasting will often be underestimated using the currently recommended incidence correction factor of 1.6. Application of updated incidence correction factors represents a simple way to improve programme planning when incidence data are not available and could inform the approach to burden estimation.

**Key questions**

**What is already known?**

- Severe wasting is an important underlying cause of mortality and morbidity among children worldwide.
- Addressing this serious health challenge requires accurate burden estimates to inform policy decisions and action.
- Currently, global estimates are only available for the number of children with severe wasting at a given point in time which is generated from national-level surveys that consider prevalent but not incident cases.
- There are no global estimates that account for children who are affected by and need treatment for severe wasting over the entire year.
- To improve programme planning, application of a single incidence correction factor of 1.6 is currently recommended as a practical approach in all settings where there are data on prevalent but not incident cases.
- The currently recommended incidence correction factor was estimated using two historical cohorts.

**What are the new findings?**

- We estimated country-specific and region-specific incidence correction factors using secondary data from 352 sites in 20 countries.
- We found that incidence correction factors varied widely within and between countries and that using the currently recommended single incidence correction factor of 1.6 will underestimate the burden of severe wasting in many settings where the burden of severe wasting is high.

**Key questions**

**What do the new findings imply?**

- Our findings underscore the need to consider incident cases when estimating the full burden of severe wasting in a given year and reaffirm the importance of severe wasting as a critical factor in child health and survival.
- The full burden of severe wasting, including incident cases, must be considered by governments and international donors to global health initiatives if effective action is to be taken to mitigate the deleterious effects of severe wasting on young children.
- High-quality and timely data are critical for accurate estimation to guide priority setting and resource allocation within the child health and survival sector.
- It is therefore essential for countries to strengthen data and information systems and for technical bodies to develop practical methodologies to define and monitor severe wasting incidence.
- Due to the variability of estimates, context-specific incidence correction factors should be applied for programprogramme planning only when other longitudinal data are not available.

## Introduction

Health system priorities should be aligned to the populations they serve. Policy-makers must therefore understand their country’s health burdens and how they shift over time.^1^ This involves estimating the number of individuals affected by a variety of illnesses, allowing decision-makers to compare trends over time and the relative burden of each in order to allocate resources effectively. Wasting, a form of acute malnutrition characterised by a loss of muscle and fat mass, increases a child’s risk of infection and death, decreases their ability to learn and makes them less productive later in life.^2–4^ Accurate estimates of wasting incidence are essential to ensure that wasting is appropriately prioritised relative to other causes of child mortality and morbidity and that sufficient resources are allocated for its treatment.

The latest United Nations (UN) estimates suggest that 47 million children under 5 years of age globally were wasted, and over 14 million severely wasted in 2019.^5^ These annual UN estimates, however, were based on national-level prevalence data alone and do not reflect the cumulative (incident) cases that occur in a year.^6 7^ As wasting is a relatively short-term condition and is affected by seasonality, estimates based on incidence are needed to support appropriate programme planning at country level and allocation of resources by the global community.

To improve programme planning, practical methods that account for both prevalent and incident cases have been endorsed by the UNICEF, the Global Nutrition Cluster and implementing partners.^8^ Current guidance offers a standardised approach to calculate the number of children in need of treatment for severe wasting by applying an incidence correction factor to prevalence estimates. One single incidence correction factor equal to 1.6 has been recommended for use everywhere.^9^ Recent work, however, suggests that the incidence correction factor may be larger than initially estimated and vary by time and place.^10–14^ The primary objective of the present analysis was to estimate incidence correction factors across a range of settings to improve programme planning and inform the approach to burden estimation for severe wasting.

## Methods

### Epidemiological framework to estimate the burden of severe wasting with an incidence correction factor

The burden of severe wasting, defined as the total number of cases from time t_0_ to t_1_, is expressed as the sum of prevalent cases at t_0_ and incident cases occurring between t_0_ and t_1_ (Equation 1).^15^ Prevalence estimates are relatively easy to obtain using cross-sectional prevalence surveys. In contrast, estimates of incidence require longitudinal follow-up of a cohort and are more costly and time-consuming to obtain. In the absence of incidence data, a practical method to improve estimation of the disease burden of severe wasting has been proposed.^8^ This method, endorsed by the UNICEF, the Global Nutrition Cluster and the implementing partners, applies an incidence correction factor to available prevalence estimates to account for unobserved incident cases for programme planning purposes.

The derivation of the incidence correction factor is based on the simple mathematical relationship between incidence, prevalence and average duration of an episode of illness.^16 17^ Assuming prevalence is low and incidence and duration of the illness do not vary over time, incidence can be approximated as prevalence multiplied by the inverse of the average duration of an episode of illness (Equations 2-3). The inverse of the average duration of an episode (K_t_) is known as the incidence correction factor, as it is shown here to ‘correct’ or be multiplied by an estimate of prevalence to arrive at an approximation of incidence (Equation 4).

(Eq. 1) $$burden=populationsize\times \left[prevalence_{t0}+incidence_{t1-t0}\right]$$

(Eq. 2) $$prevalence_{t0}=incidence_{t1-t0}*averagedurationofdisease$$

(Eq. 3) $$incidence_{t1-t0}=prevalence_{t0}*\frac{t}{averagedurationofdisease}$$

(Eq. 4) $$incidence_{t1-t0}=prevalence_{t0}*K_{t0}whereK_{t0}=\frac{t}{averagedurationofdisease}$$

and t=the period for which incidence is estimated (eg, 12 months).

In the absence of incidence data, this approximation of incidence (Equation 4) made possible using the incidence correction factor allows burden to be estimated as a function of only prevalent cases and the incidence correction factor (Equations 5-6).

(Eq. 5) $$burden=population_{6-59months}\times \left[prevalence_{t0}+prevalence_{t0}*K_{t0}\right]$$

(Eq. 6) $$burden=population_{6-59months}\times \left[prevalence_{t0}\times \left(1+K_{t0}\right)\right]$$

As indicated above, this relationship assumes a steady-state population with a low prevalence of disease and constant incidence and average duration of disease episodes.^18^ The rare disease/low prevalence assumption is likely satisfied in the case of severe wasting. The steady-state assumption may hold in a population with generally balanced in-migration and out-migration but may be more problematic if incidence is seasonal. Nevertheless, this approach has been adopted as a simple method to estimate the burden of severe wasting in the absence of incidence data and has formed the basis for UN guidance and other published work to estimate programme caseloads for severe wasting treatment programmes.^10–12^

### Data collection and extraction

Estimation of the incidence correction factor requires data on the number of (1) prevalent cases and (2) incident cases from the same geographic catchment area and time. Incident cases can be identified using longitudinal cohort data, but these data are costly and time-consuming to obtain.

Secondary data from severe wasting treatment programmes were determined to be the appropriate data source for this analysis, as they were the most readily available for the broadest geographical scope. A global call for data was issued in February 2017 to governments, UN agencies, non-governmental organisations, research institutions and other nutrition forums with a request to share existing data for the purposes of this study. We included data from sites providing four necessary data elements for the same geographical area and time period: prevalence survey data, a population size estimate, programme admission counts and a programme coverage estimate. Raw prevalence survey data including individual child data on age, sex and anthropometry were sought to allow for standardisation of the eligible population, case definitions and flagging criteria in data management and sensitivity analyses.

To obtain the number of prevalent cases (the denominator of the incidence correction factor), the proportion of severe wasting in a well-defined geographic catchment area was multiplied by the population size estimate. The target population for severe wasting was considered children aged 6–59 months.^19^ Estimates of the prevalence of severe wasting, defined as the proportion of children with severe wasting (defined as weight for height Z score (WHZ) <−3, mid-upper arm circumference (MUAC) <115 mm and/or oedema), were obtained through standardised prevalence surveys conducted in 2008 or later in which sampling was designed to be representative of the population within the catchment area. Population size, defined as the number of children aged 6–59 months living in the geographical area at the time of the prevalence survey, was taken from census estimates and adjusted for population growth since the time of the census. Population projections produced by the country’s national government were used to adjust census data to the calendar year corresponding to prevalence figures. If population projections were not available from the country’s government, country and age-specific population growth rates (10-year average from 2005 to 2015) from the UN Population Division were applied to the last available national census estimate.^20^ When population estimates were reported for children 0–59 months, 90% of this number was taken to approximate the number of children aged 6–59 months.

To obtain the number of incident cases (the numerator of the incidence correction factor) in the absence of longitudinal data, the number of children newly admitted for treatment of severe wasting during a 12-month period was multiplied by the inverse of estimated programme coverage. Programme admissions data represented all admissions in the same geographic catchment area of the prevalence survey during any 12 consecutive months including the date of the prevalence survey. Programme coverage estimates were obtained from standardised coverage surveys and defined as the proportion of children with severe wasting in the geographic catchment area and time period who received treatment among all severe wasting cases that occurred in that area and time (ie, point coverage).^21^ When multiple coverage surveys were available, the unweighted mean of all coverage estimates was used.

### Statistical analysis

Individual incidence correction factors were calculated as the number of incident cases divided by the number of prevalent cases (extension of Equation 4). 95% CIs for incidence correction factors were obtained using Monte Carlo sampling from the 95% CIs for prevalence and coverage estimates (1000 draws from a normal distribution fit to each half of the CI) to allow for uncertainty surrounding these point estimates.^22^

Estimates were aggregated to the national, regional and overall levels using inverse-variance weighted random-effects meta-analysis to account for the precision of individual incidence correction factor estimates.^23^

Stratified analyses were conducted by season of the prevalence survey, Integrated Food Security Phase Classification (IPC),^24^ presence of moderate wasting treatment programmes, presence of an acute emergency as defined by UNICEF country offices, and the wasting case definition used by the severe wasting treatment programme (eg, WHZ and/or MUAC). Heterogeneity of estimates was assessed using Cochrane’s Q statistic.^25^

Sensitivity analyses were conducted to better understand the influence of data quality on estimation. Planned analyses applied the following exclusion criteria: (1) extreme anthropometric measures in the prevalence survey data defined as WHZ <-5 or WHZ >3 SD below or above the reference median^26^; (2) coverage estimates that did not exactly match the place and time of corresponding prevalence and admissions data; (3) prevalence surveys assessed by the authors to not be a representative sample of the geographical area (eg, where sampling was constrained in the field due to insecurity); (4) admissions data that did not disaggregate readmissions from total new cases; (5) surveys with an implausible severe wasting prevalence, defined as <0.135%, the expected proportion with WHZ <-3 in a population of children with no environmental or economic constraints to growth in their homes; (6) implausible outlier values of the incidence correction factor according to expert consensus (K<1) and Tukey’s criteria, defined as values falling more than 1.5 times the length of the IQR above the third quartile^27^; and (7) prevalence surveys of poor data quality, defined using Standardised Monitoring and Assessment of Relief and Transitions (SMART) plausibility criteria for age ratio (χ^2^ p value ≤0.001 for 6–29 months vs 30–59 months), SD for WHZ ≥1.2 or ≤0.8, and digit preference score for MUAC and weight >20.^26^

### Patient and public involvement

It was not possible or appropriate to involve participants or the public in the design, conduct, reporting or dissemination plans of this secondary analysis.

## Results

### Data received

**Figure 1:**
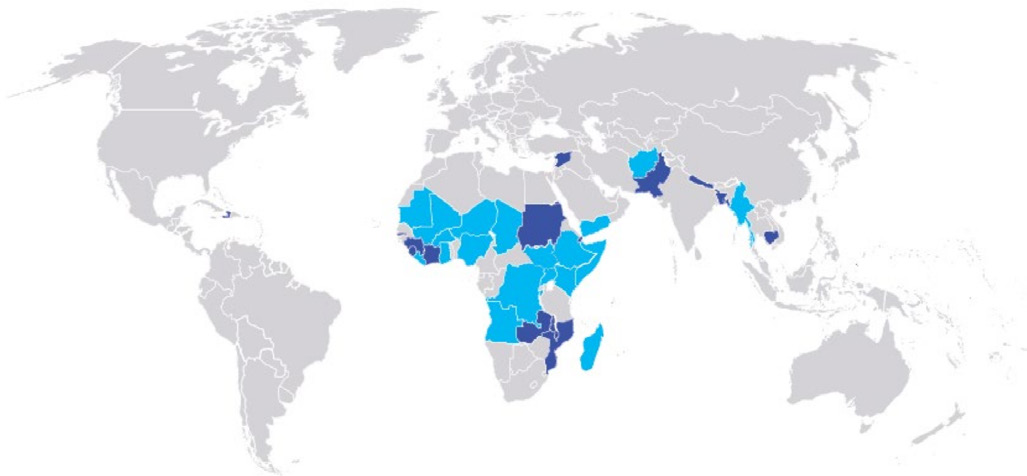
Countries providing data. Light blue represents countries that provided data appropriate for analysis, while purple represents countries providing data that were not analysed.

**Figure 2:**
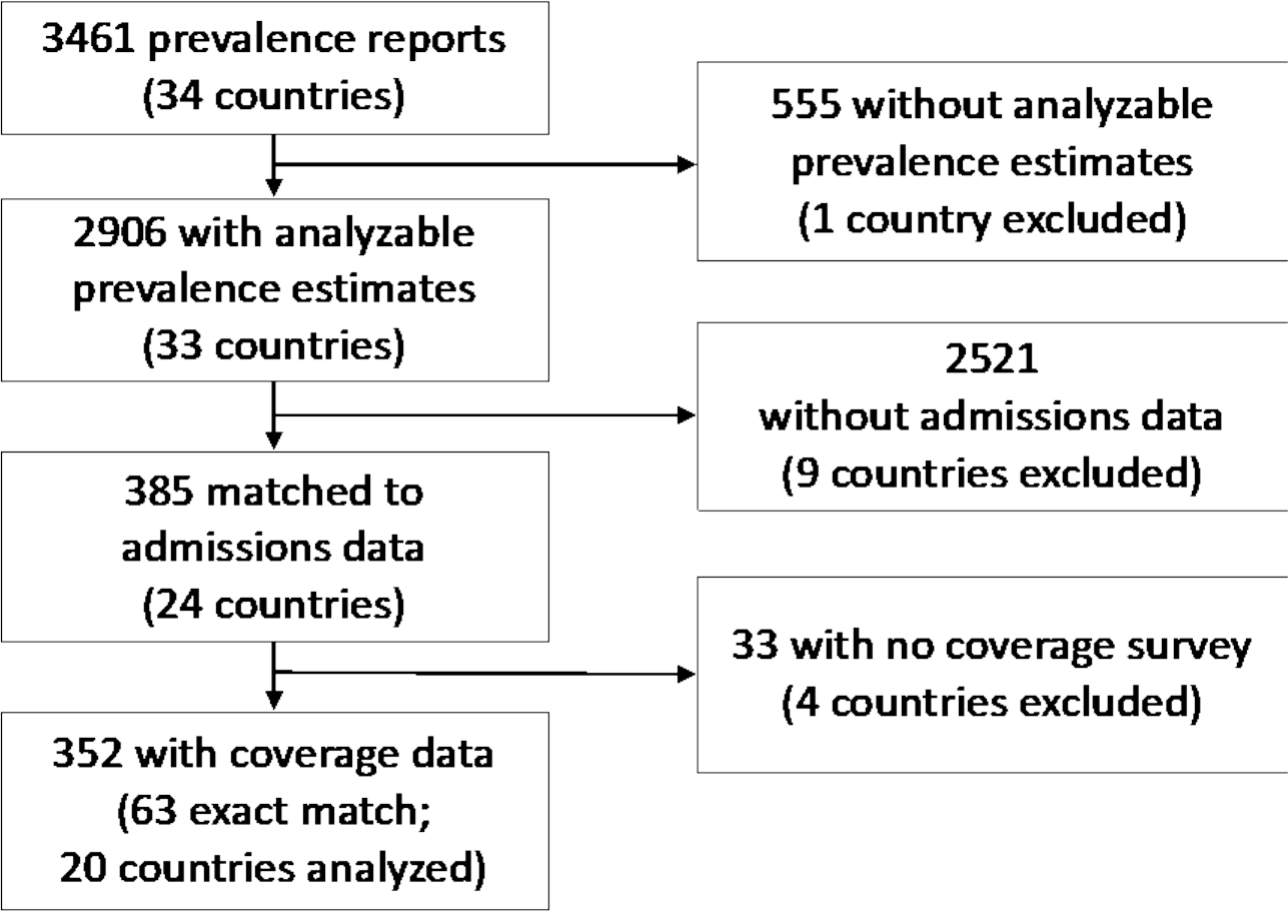
Data received, excluded and analysed.

We received data from 3461 unique sites in 34 countries (figure 1). Prevalence surveys were the most common type of data received, of which raw data were available for 84% (n=2906/3461) of sites. Programme admissions data were matched by time and place to 13% (n=385) of prevalence surveys with raw data. Among sites with matched prevalence and admissions data, 264 coverage surveys were received, of which 83% (n=218) were used in the analysis and 24% (n=63) were exactly matched to prevalence and admissions data for estimation (figure 2, online supplemental tables 1 and 2).

10.1136/bmjgh-2020-004342.supp1 Supplementary data

### Incidence correction factor estimation

A total of 352 incidence correction factors for severe wasting were calculated for 20 countries (full listing available in online supplemental table 3). Table 1 presents incidence correction factor estimates aggregated by country, region and all sites studied. The number of estimates per country varied substantially, with an average of 18 per country and a range of 1 (Angola, Burundi) to 69 (Nigeria). National-level incidence correction factor estimates ranged from 1.3 (Nigeria) to 30.1 (Burundi), regional-level estimates ranged from 2.3 (South Asia) to 12.7 (East Asia and Pacific) and the overall estimate was 3.5 (95% CI 3.1, 3.9).

**Table 1:** Summary estimates of the incidence correction factor

Geographical area	N	K	95% CI	P value*
All available	352	3.5	(3.1 to 3.9)	<0.001
Western and Central Africa	197	3.6	(3.0 to 4.3)	<0.001
Burkina Faso	31	4.0	(3.4 to 4.6)	<0.001
Chad	26	14.7	(8.8 to 2.5)	<0.001
Democratic Republic of Congo	3	4.9	(1.9 to 12.6)	<0.001
Ghana	3	1.9	(1.0 to 3.9)	0.40
Liberia	15	5.9	(3.2 to 11.1)	<0.001
Mali	32	4.6	(3.7 to 5.6)	<0.001
Mauritania	2	5.0	(0.9 to 26.4)	0.002
Niger	16	8.4	(6.2 to 11.3)	<0.001
Nigeria	69	1.3	(1.0 to 1.6)	<0.001
Eastern and Southern Africa	99	3.7	(3.1 to 4.3)	<0.001
Angola	1	6.4	(3.1 to 14.5)	–
Burundi	1	30.1	(7.0 to 106.4)	–
Ethiopia	29	6.7	(5.3 to 8.4)	<0.001
Kenya	27	2.3	(1.6 to 3.2)	<0.001
Madagascar	8	8.5	(6.7 to 10.8)	0.13
Somalia	2	9.5	(0.7 to 134.6)	<0.001
South Sudan	5	2.6	(1.8 to 3.8)	0.015
Uganda	26	2.4	(1.9 to 2.9)	<0.001
Middle East and North Africa	35	2.8	(2.3 to 3.4)	<0.001
Yemen	35	2.8	(2.3 to 3.4)	<0.001
South Asia	16	2.3	(1.4 to 3.9)	<0.001
Afghanistan	16	2.3	(1.4 to 3.9)	<0.001
East Asia and Pacific	5	12.7	(5.7 to 28.4)	<0.001
Myanmar	5	12.7	(5.7 to 28.4)	<0.001

*P value for Cochrane’s Q statistic assessing heterogeneity between K estimates within a geographical area.

There was important variation within countries and regions but no significant variation by season of the prevalence survey, presence of treatment programmes for moderate wasting or presence of an acute emergency. In sub-Saharan Africa, estimates of the incidence correction factor were significantly higher in contexts with greater food insecurity as defined by IPC phase: 3.4 (95% CI 2.8 to 4.0) in IPC Phase 1–2 vs 5.9 (95% CI 2.5 to 14.2) in IPC Phase 3–4 in Western and Central Africa and 3.2 (95% CI 2.5 to 4.0) in IPC Phase 1–2 vs 4.3 (95% CI 3.4 to 5.5) in IPC Phase 3–4 in Eastern and Southern Africa (online supplemental table 4). Data received did not allow for investigation of variation by child age or wasting case definition.

Extensive sensitivity analyses were conducted to understand the influence of data quality on estimates of the incidence correction factor. Overall, more restrictive analyses removing data of potentially poor quality resulted in larger estimates of the incidence correction factor (table 2). Removing implausible values of the incidence correction factor (eg, K<1 as per expert consensus and K>15.2 as per Tukey’s criteria) did not materially impact the overall result (3.6, 95% CI 3.4 to 3.9 in sensitivity analysis excluding implausible values versus 3.5, 95% CI 3.1 to 3.9 in the full analysis).

**Table 2:** Summary of sensitivity analyses on overall incidence correction factor

	N	K	95% CI
Base case	352	3.5	(3.1 to 3.9)
Sensitivity analyses			
Exclude anthropometric outliers by WHO method	352	3.7	(3.3 to 4.1)
Exclude anthropometric outliers by SMART method	352	4.7	(4.2 to 5.3)
Exclude coverage surveys without exact match by time and place	63	4.5	(3.7 to 5.5)
Exclude non-representative prevalence surveys	208	4.7	(4.1 to 5.3)
Exclude admissions data containing possible readmissions	114	4.3	(3.7 to 5.0)
Exclude implausible values of the incidence correction (K<1 by expert consensus or K>15.2 by Tukey’s criteria)	284	3.6	(3.4 to 3.9)
Exclude low quality prevalence surveys by SMART criteria	258	3.8	(3.4 to 4.4)

SMART, Standardised Monitoring and Assessment of Relief and Transitions.

## Discussion

Data on the number of incident cases of severe wasting in a given year remain a key requirement for priority setting and resource allocation at the global and national levels. Current approaches to estimate burden that account for prevalent but not incident cases are suboptimal, although practical methods to guide programme planning have been endorsed by UNICEF and partners to address current weaknesses. These methods recommend use of an incidence correction factor to account for both prevalent and incident cases in the absence of longitudinal data but provide one single incidence correction for use in all settings equal to 1.6. This study represents the most extensive analysis of incidence correction factors for severe wasting, providing 352 new estimates from 20 countries. Due to the variability of estimates, context-specific incidence correction factors should be applied for programme planning only when other longitudinal data are not available and the overall plausible estimate of 3.6 (95% CI 3.4 to 3.9) used only in the absence of other context-specific data.

Although original guidance acknowledged that the incidence correction factor could vary across time and place, a single incidence correction factor of 1.6 was recommended for use in programme planning in settings that lacked additional data. This estimate was derived from longitudinal data from children followed up in two Africa settings between 1983 and 1992,^9^ a period before the community-based management of wasting and scale-up of immunisation programmes and treatment for diarrhoea, malaria and other infectious diseases. Further, frequency of anthropometric surveillance in those cohorts was limited to 3-month or 6-month intervals, which could have resulted in an overestimation of the duration of illness if short episodes were not considered resolved until the next assessment and/or underestimated wasting incidence if short illnesses that started and resolved between measurements were missed altogether. In line with previous findings,^10–14 28^ our estimates suggest the incidence correction factor for severe wasting may be significantly higher than originally proposed and the burden of severe wasting underestimated in many settings. On average, our results of a plausible overall incidence correction factor of 3.6 (95% CI 3.4 to 3.9) suggest that the overall burden could be underestimated by 4.6 times using prevalence data alone and by 1.8 times using the currently recommended single incidence correction factor of 1.6 (online supplemental table 4).

This study was comprehensive in its search for secondary data, producing the most extensive analysis of incidence correction factors for severe wasting to date. Our standardised approach to data extraction and analysis facilitated comparison of a large number of estimates and examination of potential variation across countries, regions and contextual factors for the first time. We anticipated that poor data quality could bias estimates of the incidence correction factor, potentially in either direction. To better understand the influence of data quality in this study, we conducted seven planned sensitivity analyses, which suggested that poor quality data as assessed in this study may not materially alter the overall estimate but more often underestimated the incidence correction factor and burden of severe wasting.

However, this analysis has limitations. A major limitation is the scarcity of data from Asia, Europe and the Americas, which would be needed to inform estimation at the global and regional levels. Additionally, there were important exclusions of data from sites unable to provide all four required data elements for the same geographical place and time (eg, prevalence survey not aligned to dates of admission data or missing coverage estimates). This limitation resulted in a loss or misalignment of secondary data for analysis and highlights the need for high-quality data that are aligned in place and time. Further, we note that 19% (n=68/352) of incidence correction factors estimated in this analysis could be considered implausible (eg, K<1 by expert consensus or K>15.2 by Tukey’s criteria), reflecting varying levels of quality and consistency of the secondary data received relating to prevalence, population size, programme admission and programme coverage. Another limitation is that the mathematical approach to estimate burden using an incidence correction factor, although based on epidemiological principles^16 18^ and endorsed by UNICEF and partners for programme planning purposes,^15^ has not yet been validated for burden estimation of severe wasting. Further technical consultation should be prioritised to update the methodology underlying burden estimation to insure both prevalent and incident cases are accounted for. Use of incidence correction factors to do so would benefit from triangulation with longitudinal cohort data, a gold standard for estimating incidence that does not suffer from the same limitations as routine programme data. Although incidence observed within a prospective cohort may be reduced due to treatment provided for obvious ethical reasons, consistency of incidence correction factors derived from prospective cohort data with those from routine programme data from the same setting would provide reassurance for continued use and endorsement of the more practical approach involving the incidence correction factor. Inconsistency of estimated incidence correction factors would improve our understanding of the limitations of routine programme data and inform development of additional checks that could be applied if such data are to be used. Finally, this analysis focused on severe wasting among children aged 6–59 months. Corresponding estimates for moderate wasting and children 0–5 months of age were not undertaken due to data unavailability at the time of analysis. Estimates herein are not recommended for direct application to these groups, and similar research should be undertaken to develop the relevant incidence correction factors for these children.

Moving forward, all countries should be empowered to improve their estimates of the burden of severe wasting (accounting for both prevalent and incident cases) to inform priority setting, programme planning and resource allocation. Current approaches to estimate burden that rely on prevalence surveys alone^6 7^ underestimate the importance of severe wasting as a factor in child health and survival. Application of an appropriate incidence correction factor represents a simple way to consider incident cases so that prevalence-based burden estimates remain relevant in the absence of incidence data. Our experience suggests that the quality of routine programme data may present a significant challenge to the generation of new incidence correction factors and that contextual information, such as food insecurity, may be informative to consider in their generation and application. In this analysis, misalignment and poor formatting of routine data collection rendered some existing data unsuitable for analysis, while data elements such as coverage surveys were conspicuously missing in many settings (figure 1). Programmatic and contextual information that is not routinely or not completely collected, however, may be feasibly generated through national health information systems. This will demand technical expertise, organised health systems and financial resources, which should be mobilised now rather than later.

We caution that application of the incidence correction factor estimates produced herein is not straightforward and will require thoughtful consideration and communication. Detailed guidance is forthcoming and intended to provide practical guidance on how to appropriately develop and apply incidence correction factor estimates in specific contexts.^29^ Due to the variability of estimates, context-specific review of data and individual estimates is recommended for programme planning. Application of a single national-level incidence correction factor in a country or extrapolation of an incidence correction factor from one country to another could lead to problems, as correction factors appear to vary within and across countries and by contextual factors, such as food insecurity. Estimates from this analysis should be selectively applied in programme planning only when other longitudinal data and context-specific estimates are not available. Using data from a few areas and generalising to an entire country could introduce bias if substantial spatial differences exist. Drivers of variability require careful consideration before generalisation.^30^ Comparability between countries requires uniformity in case definitions and standard care, which could be improving with adoption of the updated WHO guidelines for the management of severe wasting and UNICEF guidelines to develop a core set of nutrition data collected through national health information systems.

To effectively inform priority setting and resource allocation at the national and global levels, estimates of annual incidence for severe wasting are needed. As the world moves to reduce the burden of child wasting, we must improve our ability to measure the annual incidence of this condition and empower each country to plan, implement programmes and track progress towards this ambitious goal. In the absence of incidence data, incidence correction factors offer a simple way to improve programme planning and inform the approach to burden estimation for severe wasting. Estimates of the incidence correction factor vary widely, but this study provides a starting point on which to build further work to generate additional estimates and ultimately develop practical methodologies to define and monitor severe wasting incidence.
