# Supplementary material for: Improving estimates of the burden of severe wasting: analysis of secondary prevalence and incidence data from 352 sites
Source: BMJ Glob Health. 2021 Mar 2;6(3):e004342. doi: 10.1136/bmjgh-2020-004342 (PMC7929878; doi:10.1136/bmjgh-2020-004342)
Supplement: Supplementary data [file bmjgh-2020-004342supp001.pdf]

Supplementary Online Material

Supplementary Table 1. Program data received from 34 countries for analysis of incidence correction factors

Country	N Prev	N Prev with raw data	N matched Prev, Pop, and Adm	N Cov	N Prev, Pop, Adm, and exact Cov match	Data analyzable
**All**	3461	2905	385	264	63	---
**Western and Central Africa**	1442	1173	199	165	46	---
Burkina Faso	199	169	31	24	5	Yes
Chad	275	214	26	28	8	Yes
Democratic Republic of the Congo	217	216	3	12	1	Yes
Gambia	2	2	2	0	0	No
Ghana	33	23	3	6	3	Yes
Guinea	24	24	0	0	0	No
Ivory Coast	52	49	0	3	0	No
Liberia	36	36	15	1	0	Yes
Mali	87	65	32	31	10	Yes
Mauritania	130	89	2	4	1	Yes
Niger	140	83	16	27	3	Yes
Nigeria	188	144	69	28	15	Yes
Sierra Leone	59	59	0	1	0	No
**Eastern and Southern Africa**	1535	1309	130	65	10	---
Angola	1	1	1	2	1	Yes
Burundi	25	25	1	1	0	Yes
Djibouti	14	14	6	0	0	No
Ethiopia	295	270	29	17	2	Yes
Kenya	344	168	27	26	6	Yes
Madagascar	12	12	8	1	1	Yes
Malawi	35	35	19	0	0	No
Mozambique	38	37	6	0	0	No
Somalia	521	521	2	12	0	Yes
South Sudan	166	144	5	3	0	Yes
Uganda	70	69	26	2	0	Yes
Zambia	14	14	0	1	0	No

**South Asia**	156	131	16	16	2	---
Afghanistan	71	65	16	9	2	Yes
Bangladesh	43	42	0	2	0	No
Nepal	25	12	0	2	0	No
Pakistan	17	12	0	3	0	No
**Middle East and North Africa**	208	176	35	9	1	---
Sudan	137	135	0	8	0	No
Syria	1	0	0	0	0	No
Yemen	70	41	35	1	1	Yes
**East Asia and Pacific**	45	42	5	7	4	---
Myanmar	45	42	5	7	4	Yes
**Americas**	75	74	0	2	0	---
Haiti	75	74	0	2	0	No

Abbreviations: Prev, prevalence survey; Pop, population estimate; Adm, program admissions data; Cov, coverage survey

Supplemental Table 2. Coverage survey estimates

Country	Directly matched with prevalence data	Coverage survey type	Coverage Date	Coverage Location	Coverage Point Estimate	LCL	UCL
Afghanistan	Yes	SQUEAC	3/1/2015	Paktya province; Chamkani, Ahmad abad, and Gardez districts	0.533	0.425	0.644
Afghanistan	Yes	SQUEAC	4/1/2015	Nangarhar Province; Jalalabad, Kama, and Behsud districts	0.526	0.416	0.634
Afghanistan	No	SQUEAC	5/1/2015	Laskargah, Nad-Ali, and Nawa districts of Helmand Province	0.239	0.141	0.336
Afghanistan	No	SQUEAC	5/1/2015	Khandahar City, Khandahar Province	0.407	0.323	0.491
Angola	Yes	SLEAC	4/20/2017	Huila and Cunene Provinces	0.262	0.139	0.420
Burkina Faso	No	SQUEAC	2/1/2010	DS Diapaga, Est Region	0.206	0.110	0.301
Burkina Faso	Yes	SQUEAC	3/1/2011	DS Diapaga, Est Region	0.176	0.078	0.316
Burkina Faso	Yes	SQUEAC	11/1/2011	DS Seguenega, Nord Region	0.468	0.318	0.617
Burkina Faso	Yes	SQUEAC	2/1/2012	DS Fada, Est Region	0.191	0.081	0.302
Burkina Faso	Yes	SQUEAC	3/1/2012	DS Diapaga, Est Region	0.321	0.207	0.436
Burkina Faso	Yes	SQUEAC	6/1/2012	DS Dori, Sahel Region	0.385	0.284	0.485
Burkina Faso	Yes	SQUEAC	11/1/2012	DS Pama, Est Region	0.346	0.217	0.475
Burkina Faso	Yes	SQUEAC	12/15/2012	DS Djibo, Sahel Region	0.473	0.357	0.589
Burkina Faso	Yes	SQUEAC	2/15/2013	DS Yako, Nord Region	0.308	0.202	0.413
Burkina Faso	Yes	SQUEAC	5/15/2013	DS Bogande, Est Region	0.302	0.197	0.406
Burkina Faso	Yes	SQUEAC	5/15/2013	DS Manni, Est Region	0.261	0.156	0.366
Burkina Faso	Yes	SQUEAC	11/15/2013	DS Gorom-Gorom, Sahel Region	0.375	0.282	0.468
Burkina Faso	Yes	SQUEAC	11/15/2013	DS Sebba, Sahel Region	0.461	0.354	0.567
Burkina Faso	Yes	SQUEAC	1/15/2014	DS Fada N'Gourma, Est Region	0.494	0.385	0.602
Burkina Faso	Yes	SQUEAC	2/1/2014	DS Ouargaye, Centre Est Region	0.218	0.132	0.305
Burkina Faso	Yes	SQUEAC	2/1/2014	DS Zorgho, Plateau Central Region	0.533	0.410	0.657
Burkina Faso	Yes	SQUEAC	2/15/2014	DS Manni, Est Region	0.524	0.429	0.619
Burkina Faso	Yes	SQUEAC	2/15/2014	DS Koudougou, Centre Ouest Region	0.615	0.512	0.717

Country	Directly matched with prevalence data	Coverage survey type	Coverage Date	Coverage Location	Coverage Point Estimate	LCL	UCL
Burkina Faso	Yes	SQUEAC	2/15/2014	DS Bogande, Est Region	0.364	0.272	0.456
Burkina Faso	Yes	SQUEAC	2/15/2014	DS Diapaga, Est Region	0.436	0.340	0.531
Burkina Faso	Yes	SQUEAC	2/15/2014	DS Reo, Centre Ouest Region	0.389	0.281	0.497
Burkina Faso	Yes	SQUEAC	2/15/2014	DS Nanoro, Centre Ouest Region	0.350	0.222	0.478
Burkina Faso	Yes	SQUEAC	9/26/2015	DS Gourcy, Nord Region	0.488	0.381	0.595
Burkina Faso	Yes	SQUEAC	3/1/2017	DS Fada N'gourma, Est Region	0.435	0.339	0.531
Burundi	No	CSAS	2/1/2013	Muramvya Province	0.425	0.354	0.494
Chad	No	SQUEAC	12/1/2010	Région de Kanem; District sanitaire de Mao	0.271	0.165	0.394
Chad	No	SQUEAC	9/1/2011	Région de Kanem; Districts Sanitaires de Mao et de Mondo	0.364	0.269	0.471
Chad	No	SQUEAC	11/1/2011	Région de Bahr el Ghazel; District Sanitaire de Moussoro	0.341	0.221	0.486
Chad	No	SQUEAC	8/1/2012	Région de Kanem; Districts Sanitaires de Mao et de Mondo	0.376	0.276	0.485
Chad	No	SQUEAC	9/1/2012	Région du Bahr el Gazal Sud, Moussoro	0.291	0.206	0.394
Chad	No	SQUEAC	10/1/2012	Région de Bahr el Ghazel, DS de Moussoro	0.341	0.221	0.486
Chad	Yes	SQUEAC	1/1/2013	Région de Kanem; Districts de Mao et Mondo	0.374	0.282	0.478
Chad	Yes	SQUEAC	3/1/2013	Région de Wadi Fira, DS d'Iriba	0.364	0.269	0.460
Chad	Yes	SQUEAC	3/1/2013	Région de Sila, DS d'Amdam	0.443	0.339	0.547
Chad	Yes	SQUEAC	4/1/2013	Région de Bahr el Ghazel - Sud	0.415	0.279	0.552
Chad	Yes	SQUEAC	4/1/2013	Région de Batha, DS d'Ati et d'Oum Hadjer	0.197	0.109	0.285
Chad	No	SQUEAC	9/1/2013	Région de Bahr el Gazal, Sud	0.377	0.290	0.473
Chad	Yes	SQUEAC	9/1/2013	Région de Kanem, districts de Mao et de Mondo	0.353	0.255	0.467
Chad	Yes	SQUEAC	10/1/2013	Région de Sila, DS de Goz Beida	0.376	0.260	0.491
Chad	Yes	SQUEAC	11/1/2013	Région de Salamat, DS d'Aboudeia	0.431	0.326	0.536
Chad	Yes	SQUEAC	12/1/2013	Région de Hadjer Lamis, DS de Massaguet	0.460	0.357	0.563
Chad	Yes	SQUEAC	1/1/2014	Région de Hadjer Lamis, DS de Bokoro	0.346	0.248	0.443
Chad	Yes	SQUEAC	8/1/2014	Région de Guera, DS de Bitkine	0.276	0.167	0.385

Country	Directly matched with prevalence data	Coverage survey type	Coverage Date	Coverage Location	Coverage Point Estimate	LCL	UCL
Chad	Yes	SQUEAC	9/1/2014	Région de Guera, DS de Mangalmé	0.449	0.365	0.533
Chad	No	SQUEAC	3/1/2015	Région de Bahr el Gazal, DS de Chaddra et Moussoro	0.617	0.509	0.724
DRC	No	SLEAC	7/1/2012	Territoires de Bagata, Popokabaka, KasongoLunda (BandunduLubutu (Maniema) et Lodja (Kasaï-Oriental)	0.100	0.072	0.133
DRC	No	SQUEAC	1/15/2013	Kisantu	0.265	0.166	0.365
DRC	No	SQUEAC	2/15/2013	Opala	0.315	0.196	0.433
DRC	No	SQUEAC	2/15/2013	Bafwasende	0.405	0.260	0.549
DRC	No	SQUEAC	6/1/2013	Manono	0.499	0.380	0.618
DRC	No	SQUEAC	10/1/2013	Opienge	0.416	0.291	0.541
DRC	No	SQUEAC	3/15/2014	Mosango	0.327	0.219	0.435
DRC	No	SQUEAC	12/1/2014	Kirotshe	0.323	0.231	0.416
DRC	No	SQUEAC	2/1/2015	Kirotshe	0.392	0.292	0.492
DRC	Yes	SQUEAC	3/31/2016	Kalomba	0.379	0.282	0.475
Ethiopia	No	SQUEAC	1/1/2010	Arsi Negele	0.200	0.090	0.310
Ethiopia	No	SQUEAC	9/1/2012	Dollo Ado Camp	0.613	0.473	0.753
Ethiopia	No	SQUEAC	7/1/2013	Meyu Muluke	0.652	0.543	0.762
Ethiopia	No	SQUEAC	8/1/2013	Dollo Ado Camp	0.887	0.815	0.960
Ethiopia	No	SQUEAC	9/15/2014	Bati	0.416	0.272	0.560
Ethiopia	No	SQUEAC	11/1/2014	Adadle Woreda, Somali	0.538	0.441	0.635
Ethiopia	Yes	SQUEAC	2/2/2015	Sekota	0.422	0.300	0.543
Ethiopia	No	SQUEAC	6/26/2015	Boloso Sore	0.431	0.332	0.529
Ethiopia	No	SQUEAC	9/1/2016	Hintalo Wajirat	0.457	0.324	0.599
Ethiopia	No	SQUEAC	1/1/2017	Sahreti Samre Woreda, Tigray	0.528	0.433	0.648
Ethiopia	No	SQUEAC	2/1/2017	Abaya Woreda, Oromia	0.377	0.275	0.487
Ethiopia	No	SQUEAC	2/1/2017	Abergele Woreda, Amhara	0.355	0.259	0.450

Country	Directly matched with prevalence data	Coverage survey type	Coverage Date	Coverage Location	Coverage Point Estimate	LCL	UCL
Ethiopia	No	SQUEAC	4/1/2017	Shashamane Woreda, Oromia	0.492	0.392	0.593
Ethiopia	Yes	SQUEAC	4/1/2017	Miesso Woreda, Oromiya	0.509	0.378	0.637
Ethiopia	No	SQUEAC	5/1/2017	Deder Woreda, Oromia	0.521	0.383	0.660
Ethiopia	No	SQUEAC	6/1/2017	Bena Woreda	0.358	0.234	0.482
Ethiopia	No	SQUEAC	6/1/2017	Aleto Chuko Woreda, SNNPR	0.543	0.418	0.668
Ghana	Yes	SLEAC	3/1/2014	Tamale Metro district, Northern Region	0.026	0.001	0.138
Ghana	Yes	SLEAC	3/1/2014	Kassena Nankana district, Upper East Region	0.067	0.002	0.319
Ghana	Yes	SLEAC	3/1/2014	Wa East district, Upper West Region	0.071	0.002	0.339
Ghana	Yes	SLEAC	3/1/2014	Tolon district, Northern Region	0.083	0.002	0.385
Ghana	Yes	SLEAC	3/1/2014	Bawku West district, Upper East Region	0.450	0.231	0.685
Ghana	Yes	SLEAC	3/1/2014	Jirapa district, Upper West Region	0.083	0.002	0.385
Kenya	Yes	SQUEAC	6/1/2012	West Pokot	0.335	0.230	0.440
Kenya	Yes	SQUEAC	10/26/2012	Merti	0.335	0.183	0.487
Kenya	Yes	SQUEAC	12/15/2012	Laikipia	0.419	0.309	0.529
Kenya	No	SQUEAC	2/15/2013	Kisumu East	0.490	0.392	0.588
Kenya	Yes	SQUEAC	3/15/2013	Garbatulla	0.505	0.337	0.673
Kenya	No	SQUEAC	6/15/2013	Njiru	0.521	0.413	0.629
Kenya	No	SQUEAC	6/15/2013	Westlands	0.499	0.385	0.613
Kenya	No	SQUEAC	6/15/2013	Makadara	0.340	0.231	0.449
Kenya	No	SQUEAC	6/15/2013	Starehe	0.519	0.403	0.635
Kenya	No	SQUEAC	6/15/2013	Kamukunji	0.666	0.555	0.777
Kenya	No	SQUEAC	6/15/2013	Embakasi	0.310	0.199	0.421
Kenya	No	SQUEAC	6/15/2013	Kasarani	0.391	0.274	0.508
Kenya	No	SQUEAC	6/15/2013	Dagoretti	0.508	0.372	0.644
Kenya	Yes	SLEAC	7/1/2013	West Pokot	0.217	0.127	0.307

Country	Directly matched with prevalence data	Coverage survey type	Coverage Date	Coverage Location	Coverage Point Estimate	LCL	UCL
Kenya	No	SQUEAC	9/15/2013	Wajir East	0.546	0.408	0.684
Kenya	No	SQUEAC	10/6/2013	Mandera West	0.462	0.329	0.596
Kenya	No	SQUEAC	10/15/2013	Mandera Central	0.504	0.374	0.634
Kenya	No	SQUEAC	10/15/2013	Chalbi	0.202	0.078	0.326
Kenya	No	SQUEAC	10/15/2013	Wajir South	0.426	0.284	0.568
Kenya	No	SQUEAC	6/1/2014	West Pokot	0.325	0.135	0.515
Kenya	No	SQUEAC	3/18/2015	Dadaab Hagadera	0.901	0.835	0.966
Kenya	No	SQUEAC	5/28/2015	Dadaab IFO N	0.801	0.716	0.887
Kenya	No	SQUEAC	6/17/2015	Dadaab Kambioos	0.868	0.792	0.945
Kenya	No	SQUEAC	10/28/2015	East Pokot	0.457	0.310	0.605
Kenya	Yes	SQUEAC	9/1/2016	Laisamis Sub-county, Marsabit County	0.546	0.419	0.673
Kenya	Yes	SQUEAC	9/1/2016	Saku Sub-county, Marsabit County	0.530	0.384	0.676
Liberia	No	SQUEAC	2/1/2011	Monrovia	0.248	0.156	0.370
Madagascar	Yes	SLEAC	11/1/2017	District de Betioky; Région d'Atsimo Andrefana	0.287	0.209	0.376
Mali	No	SQUEAC	8/1/2011	Région de Gao	0.354	0.273	0.447
Mali	No	SQUEAC	8/1/2011	Gao	0.354	0.265	0.444
Mali	No	SQUEAC	3/1/2013	Cercle de Kita	0.249	0.146	0.393
Mali	No	SQUEAC	3/15/2013	Kita	0.249	0.123	0.375
Mali	No	SQUEAC	4/2/2013	Kati Cercle, Koulikoro Region	0.697	0.627	0.767
Mali	Yes	SQUEAC	12/15/2013	Koutiala Cercle, Sikassa Region	0.327	0.222	0.432
Mali	Yes	SLEAC	4/1/2014	Région de Kayes	0.245	0.181	0.306
Mali	Yes	SLEAC	4/1/2014	Région de Koulikoro	0.319	0.269	0.371
Mali	Yes	SLEAC	4/1/2014	Région de Mopti	0.128	0.071	0.188
Mali	Yes	SLEAC	4/1/2014	Région de Sikasso	0.235	0.183	0.288
Mali	Yes	SLEAC	4/1/2014	Région de Tombouctou	0.188	0.131	0.228

Country	Directly matched with prevalence data	Coverage survey type	Coverage Date	Coverage Location	Coverage Point Estimate	LCL	UCL
Mali	Yes	SQUEAC	4/15/2014	Nara Cercle, Koulikoro Region	0.268	0.185	0.350
Mali	Yes	SQUEAC	7/29/2014	Tominian Cercle, Segou Region	0.197	0.098	0.296
Mali	Yes	SQUEAC	8/1/2014	Kenieba Cercle, Kayes Region	0.259	0.158	0.360
Mali	Yes	SQUEAC	9/12/2014	Macina Cercle, Segou Region	0.364	0.235	0.494
Mali	Yes	SQUEAC	10/13/2014	Mopti Cercle, Mopti Region	0.463	0.360	0.566
Mali	No	SQUEAC	11/1/2014	Markala	0.273	0.191	0.355
Mali	Yes	SQUEAC	12/1/2014	Kita Cercle, Kayes Region	0.364	0.256	0.489
Mali	Yes	SQUEAC	12/1/2014	Kita Cercle, Kayes Region	0.527	0.405	0.649
Mali	Yes	SQUEAC	12/4/2014	Segou Cercle, Segou Region	0.696	0.614	0.779
Mali	Yes	SQUEAC	1/1/2015	Kati Cercle, Koulikoro Region	0.402	0.297	0.508
Mali	Yes	SQUEAC	2/6/2015	Nioro Cercle, Kayes Region	0.442	0.315	0.569
Mali	Yes	SQUEAC	3/1/2015	Yelimane Cercle, Kayes Region	0.309	0.205	0.414
Mali	Yes	SQUEAC	3/1/2015	Kayes Cercle, Kayes Region	0.211	0.113	0.310
Mali	Yes	SQUEAC	7/1/2015	Kita Cercle, Kayes Region	0.316	0.226	0.422
Mali	Yes	SQUEAC	11/15/2015	Baroueli Cercle, Segou Region	0.348	0.248	0.448
Mali	Yes	SQUEAC	11/30/2015	Kati Cercle, Koulikoro Region	0.486	0.380	0.592
Mali	Yes	SQUEAC	12/20/2015	Macina Cercle, Segou Region	0.382	0.270	0.493
Mali	No	SQUEAC	12/22/2015	Markala	0.425	0.313	0.538
Mali	Yes	SQUEAC	1/1/2017	Koro Cercle, Mopti Region	0.385	0.285	0.484
Mauritania	No	CSAS	12/1/2009	Guidimakha Region	0.293	0.161	0.455
Mauritania	No	SQUEAC	3/1/2011	Guidimakha Region	0.330	0.207	0.487
Mauritania	No	SQUEAC	5/1/2012	Guidimakha Region	0.382	0.263	0.516
Mauritania	Yes	SLEAC	11/1/2013	Gorgol Region	0.321	0.199	0.463
Myanmar	No	SQUEAC	11/30/2011	Maungdaw and Buthidaung	0.407	0.335	0.479
Myanmar	No	SQUEAC	11/30/2011	Buthidaung	0.613	0.510	0.716

Country	Directly matched with prevalence data	Coverage survey type	Coverage Date	Coverage Location	Coverage Point Estimate	LCL	UCL
Myanmar	No	SQUEAC	1/1/2014	Pauktaw IDP	0.921	0.835	1.000
Myanmar	Yes	SQUEAC	10/1/2014	Sittwe Rural	0.653	0.538	0.767
Myanmar	Yes	SQUEAC	12/1/2014	Buthidung Township	0.567	0.483	0.647
Myanmar	Yes	SQUEAC	12/1/2014	Maungdaw	0.297	0.214	0.379
Myanmar	Yes	SQUEAC	2/1/2015	Pauktaw IDP	0.821	0.716	0.927
Niger	No	SQUEAC	1/1/2012	Matameye	0.532	0.451	0.614
Niger	No	SQUEAC	5/1/2012	Keita	0.214	0.142	0.286
Niger	No	SQUEAC	2/1/2013	Mayahi	0.312	0.248	0.377
Niger	No	SQUEAC	2/1/2013	Tessaoua	0.293	0.204	0.383
Niger	No	SQUEAC	2/1/2013	Keita	0.281	0.198	0.364
Niger	No	SQUEAC	2/2/2013	N'Guigmi	0.629	0.466	0.792
Niger	No	SQUEAC	3/15/2013	Gaya	0.296	0.201	0.391
Niger	No	SQUEAC	6/15/2013	Zinder	0.480	0.377	0.584
Niger	No	SQUEAC	6/19/2013	Tera	0.527	0.417	0.638
Niger	No	SQUEAC	8/1/2013	Fillingué	0.245	0.121	0.370
Niger	No	SQUEAC	9/1/2013	Ouallam	0.240	0.166	0.314
Niger	No	SQUEAC	9/15/2013	Mirriah	0.342	0.265	0.418
Niger	No	SQUEAC	11/1/2013	Matameye	0.369	0.301	0.436
Niger	No	SQUEAC	12/1/2013	Fillingué	0.318	0.233	0.403
Niger	No	SQUEAC	12/1/2013	Agadez	0.479	0.386	0.572
Niger	No	SQUEAC	12/18/2013	Dakoro	0.639	0.568	0.710
Niger	No	SQUEAC	12/31/2013	Maine-Soroa	0.505	0.384	0.626
Niger	No	SQUEAC	1/1/2014	Illéla	0.361	0.272	0.451
Niger	No	SQUEAC	3/1/2014	Madarounfa	0.197	0.137	0.256
Niger	No	SQUEAC	7/1/2014	Tanout	0.476	0.369	0.582

Country	Directly matched with prevalence data	Coverage survey type	Coverage Date	Coverage Location	Coverage Point Estimate	LCL	UCL
Niger	Yes	SQUEAC	8/1/2014	Mirriah, Zinder	0.317	0.206	0.372
Niger	Yes	SQUEAC	11/3/2014	Tillaberi	0.528	0.426	0.629
Niger	Yes	SQUEAC	12/16/2014	Agadez	0.439	0.352	0.526
Niger	Yes	SQUEAC	11/26/2015	Tanout	0.376	0.299	0.452
Niger	Yes	SQUEAC	12/31/2015	Tillaberi	0.598	0.489	0.708
Nigeria	Yes	SQUEAC	7/29/2011	Fune, Yobe	0.330	0.238	0.422
Nigeria	Yes	SQUEAC	4/15/2013	Fune, Yobe	0.348	0.237	0.458
Nigeria	Yes	SQUEAC	5/15/2013	Baure, Katsina	0.534	0.439	0.629
Nigeria	Yes	SQUEAC	11/21/2013	Birnin Kudu, Jigawa	0.223	0.148	0.298
Nigeria	Yes	SQUEAC	11/21/2013	Gwiwa, Jigawa	0.480	0.374	0.586
Nigeria	Yes	SQUEAC	11/21/2013	Guri, Jigawa	0.504	0.399	0.610
Nigeria	Yes	SQUEAC	2/1/2014	Bakura, Zamfara	0.471	0.379	0.562
Nigeria	Yes	SQUEAC	2/1/2014	Bungudu, Zamfara	0.349	0.257	0.442
Nigeria	Yes	SQUEAC	2/1/2014	Shinkafi, Zamfara	0.470	0.363	0.578
Nigeria	Yes	SQUEAC	3/15/2014	Goronyo, Sokoto	0.143	0.079	0.208
Nigeria	Yes	SQUEAC	4/15/2014	Kalgo, Kebbi	0.305	0.206	0.403
Nigeria	Yes	SQUEAC	5/1/2014	Damaturu, Yobe	0.282	0.192	0.373
Nigeria	Yes	SQUEAC	6/1/2014	Birnin Magaji, Zamfara	0.292	0.227	0.357
Nigeria	Yes	SQUEAC	7/1/2014	Kiyawa, Jigawa	0.485	0.411	0.558
Nigeria	Yes	SQUEAC	7/1/2014	Kaita, Katsina	0.324	0.221	0.426
Nigeria	Yes	SQUEAC	9/1/2014	Katagum, Bauchi	0.195	0.127	0.262
Nigeria	Yes	SQUEAC	9/1/2014	Bichi, Kano	0.280	0.179	0.381
Nigeria	Yes	SQUEAC	9/1/2014	Fune, Yobe	0.511	0.421	0.601
Nigeria	Yes	SQUEAC	10/31/2014	Song, Adamawa	0.276	0.183	0.368
Nigeria	Yes	SQUEAC	10/31/2014	Gombe, Gombe	0.273	0.174	0.372

Country	Directly matched with prevalence data	Coverage survey type	Coverage Date	Coverage Location	Coverage Point Estimate	LCL	UCL
Nigeria	Yes	SQUEAC	11/17/2014	Binji and Wamako, Sokoto	0.823	0.755	0.891
Nigeria	Yes	SQUEAC	1/15/2015	South sokoto, Sokoto	0.395	0.298	0.493
Nigeria	Yes	SQUEAC	1/25/2015	Goronyo, Sokoto	0.146	0.092	0.201
Somalia	No	SQUEAC	3/31/2012	Galkayo Rural	0.422	0.355	0.489
Somalia	No	SQUEAC	6/15/2013	Garowe Camp	0.850	0.787	0.913
Somalia	No	SQUEAC	2/28/2014	Beledweyne Rural	0.705	0.640	0.770
Somalia	No	SQUEAC	3/11/2015	Mogadishu Urban	0.532	0.414	0.649
Somalia	No	SQUEAC	9/17/2015	Daynille district	0.534	0.412	0.653
Somalia	No	SQUEAC	10/5/2015	Baidoa district	0.424	0.337	0.519
Somalia	No	SQUEAC	10/25/2015	Dollow district	0.474	0.374	0.571
Somalia	No	SQUEAC	10/31/2015	Dhusamareb district	0.419	0.306	0.539
Somalia	No	SQUEAC	11/15/2015	Lughaya Rural	0.599	0.461	0.737
Somalia	No	SQUEAC	10/4/2017	Gabiley	0.523	0.376	0.669
Somalia	No	SQUEAC	11/9/2017	Bossaso district	0.655	0.555	0.742
Somalia	No	SQUEAC	12/1/2017	Burao district	0.534	0.411	0.653
South Sudan	No	SQUEAC	2/1/2016	Aweil East County, Northern Bahr-el-Ghazal	0.344	0.220	0.497
South Sudan	No	SQUEAC	3/1/2016	Akobo East County, Jonglei State	0.445	0.338	0.562
South Sudan	No	SQUEAC	5/1/2016	Aweil North County, Lol State	0.435	0.329	0.546
Uganda	No	SQUEAC	1/1/2015	Karamoja Subregion	0.490	0.470	0.520
Uganda	No	SLEAC	6/1/2016	Karamoja Subregion	0.198	0.122	0.294
Yemen	Yes	SQUEAC	9/1/2013	Al Qanawis, Houdeidah Gov	0.616	0.515	0.717

BMJ Publishing Group Limited (BMJ) disclaims all liability and responsibility arising from any reliance placed on this supplemental material which has been supplied by the author(s)

Supplemental Table 3. Individual K Estimates

Country	Sub-national location	Date	IPC Phase	Prevalence estimate taken during hungry season	MAM treatment available	Acute emergency	SAM treatment admissions (n)	Coverage estimate (%); [*denotes country average]	Estimated incident cases (n)	Population (n)	Prevalence (%)	K estimate
Afghanistan	Badghis province	Feb-2016	Phase 3	No	-	-	3124	42.6 (32.6, 52.6)*	7329	80772	3.2 (2.2, 4.6)	2.8 (1.9, 4.3)
Afghanistan	Balkh province	Aug-2015	-	Yes	-	-	3849	42.6 (32.6, 52.6)*	9030	217684	2.5 (1.4, 4.4)	1.6 (0.9, 3.1)
Afghanistan	Farah province	Feb-2017	-	No	-	-	2257	42.6 (32.6, 52.6)*	5295	83447	2.7 (1.8, 4.2)	2.3 (1.4, 3.8)
Afghanistan	Ghazni province	Jan-2016	Phase 2	No	-	-	6348	42.6 (32.6, 52.6)*	14893	200195	7.4 (5.9, 9.3)	1.0 (0.8, 1.4)
Afghanistan	Ghor province	Aug-2016	Phase 3	Yes	-	-	6510	42.6 (32.6, 52.6)*	15273	112384	8.4 (6.5, 10.7)	1.6 (1.2, 2.3)
Afghanistan	Helmand province; Garamser district	Mar-2015	Phase 2	No	-	-	1233	42.6 (32.6, 52.6)*	2893	17374	3.5 (2.9, 4.4)	4.7 (3.5, 6.5)
Afghanistan	Kapisa province	Oct-2016	Phase 3	No	-	-	590	42.6 (32.6, 52.6)*	1384	71790	5.5 (4.2, 7.2)	0.4 (0.2, 0.5)
Afghanistan	Khost province; Matoon (Khost), Tanya and Mandozi (Ismail khail) districts	Jun-2015	Phase 3	No	-	-	2524	42.6 (32.6, 52.6)*	5921	41828	3.3 (2.4, 4.4)	4.3 (3.0, 6.4)
Afghanistan	Laghman province; Mehterlam, Alingar, Alishing, Dawlat shah, Qarghayi districts	Dec-2015	-	No	-	-	6312	42.6 (32.6, 52.6)*	14808	70676	5.7 (4.4, 7.4)	3.7 (2.6, 5.3)
Afghanistan	Nangarhar province	Nov-2016	Phase 2	No	-	-	23629	42.6 (32.6, 52.6)*	55435	249037	1.0 (0.5, 2.1)	22.4 (10.2, 48.6)
Afghanistan	Nangarhar Province; Jalalabad, Surkhrod, Kama, Behsud, and Kuzkunar (Khewa) districts	Dec-2014	Phase 2	No	-	-	7252	52.6 (41.6, 63.4)	13787	97873	4.9 (3.6, 6.6)	2.9 (2.1, 4.2)
Afghanistan	Nuristan province; Parun, Noorgram, and Wama districts	Aug-2015	Phase 3	Yes	-	-	840	42.6 (32.6, 52.6)*	1971	9369	8.7 (6.9, 10.9)	2.4 (1.8, 3.5)

Country	Sub-national location	Date	IPC Phase	Prevalence estimate taken during hungry season	MAM treatment available	Acute emergency	SAM treatment admissions (n)	Coverage estimate (%); [*denotes country average]	Estimated incident cases (n)	Population (n)	Prevalence (%)	K estimate
Afghanistan	Paktika province; Mata Khan, Sharana, Yahakhail, Sar Hawza, and Khair kot (Zarghon Shair) districts	May-2015	Phase 3	No	-	-	3477	42.6 (32.6, 52.6)*	8157	29254	3.5 (2.5, 4.9)	8.0 (5.3, 12.1)
Afghanistan	Paktya province; Chamkani, Ahmad abad, Said karam and Gardez districts	Jun-2015	-	No	-	-	3960	53.3 (42.5, 64.4)	7430	35878	2.4 (1.6, 3.5)	8.7 (5.6, 13.2)
Afghanistan	Panjshir province	Apr-2016	Phase 2	No	-	-	212	42.6 (32.6, 52.6)*	497	24984	5.5 (4.2, 7.3)	0.4 (0.3, 0.5)
Afghanistan	Parwan province	Sep-2016	Phase 2	Yes	-	-	1835	42.6 (32.6, 52.6)*	4305	108334	7.0 (5.4, 9.1)	0.6 (0.4, 0.8)
Angola	Cubal & Ganda Municipalities	Mar-2017	-	Yes	Yes	No	3401	26.2 (13.9, 42.0)	12981	93283	2.2 (1.3, 3.6)	6.4 (3.1, 14.5)
Burkina Faso	Boucle du Mouhoun Region	Sep-2016	Phase 1	-	-	-	9058	36.7 (26.2, 47.5)*	24648	319113	2.1 (1.3, 3.2)	3.7 (2.2, 6.7)
Burkina Faso	Boucle du Mouhoun Region	Sep-2017	Phase 1	-	-	-	8467	36.7 (26.2, 47.5)*	23040	324708	1.5 (1.1, 2.1)	4.6 (3.1, 7.4)
Burkina Faso	Cascades Region	Sep-2016	Phase 1	-	-	-	3367	36.7 (26.2, 47.5)*	9162	122073	1.9 (1.0, 3.5)	4.0 (2.1, 8.0)
Burkina Faso	Cascades Region	Sep-2017	Phase 1	-	-	-	3023	36.7 (26.2, 47.5)*	8226	125204	2.2 (1.4, 3.4)	3.0 (1.8, 5.3)
Burkina Faso	Centre Est Region	Sep-2016	Phase 1	-	-	-	5010	36.7 (26.2, 47.5)*	13633	263783	1.4 (0.7, 2.6)	3.8 (1.9, 8.4)
Burkina Faso	Centre Est Region	Sep-2017	Phase 1	-	-	-	3955	36.7 (26.2, 47.5)*	10762	269167	1.8 (1.2, 2.8)	2.2 (1.4, 3.9)
Burkina Faso	Centre Nord Region	Sep-2016	Phase 1	-	-	-	9874	36.7 (26.2, 47.5)*	26868	282362	0.7 (0.3, 1.7)	13.0 (5.1, 31.5)
Burkina Faso	Centre Nord Region	Sep-2017	Phase 1	-	-	-	9455	36.7 (26.2, 47.5)*	25728	287866	1.7 (1.1, 2.7)	5.2 (3.0, 8.9)
Burkina Faso	Centre Ouest Region	Sep-2016	Phase 1	-	-	-	10066	36.7 (26.2, 47.5)*	27391	263377	1.4 (0.8, 2.6)	7.3 (3.5, 15.4)
Burkina Faso	Centre Ouest Region	Sep-2017	Phase 1	-	-	-	8605	36.7 (26.2, 47.5)*	23415	268230	1.5 (0.8, 3.0)	5.8 (2.8, 13.4)
Burkina Faso	Centre Region	Sep-2016	Phase 1	-	-	-	3544	36.7 (26.2, 47.5)*	9644	326885	1.7 (0.9, 3.5)	1.7 (0.9, 3.5)

Country	Sub-national location	Date	IPC Phase	Prevalence estimate taken during hungry season	MAM treatment available	Acute emergency	SAM treatment admissions (n)	Coverage estimate (%); [*denotes country average]	Estimated incident cases (n)	Population (n)	Prevalence (%)	K estimate
Burkina Faso	Centre Region	Sep-2017	Phase 1	-	-	-	4525	36.7 (26.2, 47.5)*	12313	336474	3.2 (1.5, 6.6)	1.1 (0.5, 2.7)
Burkina Faso	Centre Sud Region	Sep-2016	Phase 1	-	-	-	1786	36.7 (26.2, 47.5)*	4860	137958	0.2 (0.0, 1.3)	18.5 (2.2, 141.3)
Burkina Faso	Centre Sud Region	Sep-2017	Phase 1	-	-	-	1664	36.7 (26.2, 47.5)*	4528	140340	1.8 (1.1, 2.8)	1.8 (1.1, 3.1)
Burkina Faso	Countrywide	Sep-2011	Phase 2	-	-	-	54613	28.9 (17.1, 41.7)	188931	2783611	2.6 (2.3, 3.0)	2.6 (1.8, 4.5)
Burkina Faso	Countrywide	Sep-2012	Phase 1	-	-	-	91891	37.8 (26.5, 49.0)	243171	2860908	2.3 (2.1, 2.5)	3.7 (2.8, 5.4)
Burkina Faso	Countrywide	Sep-2013	Phase 1	-	-	-	104582	40.9 (30.6, 51.3)	255551	2921111	2.0 (1.6, 2.3)	4.5 (3.4, 6.4)
Burkina Faso	Countrywide	Sep-2014	Phase 2	-	-	-	116262	36.7 (26.2, 47.5)*	316363	2982797	1.8 (1.5, 2.1)	5.8 (4.3, 8.4)
Burkina Faso	Countrywide	Nov-2015	Phase 1	-	-	-	103542	48.8 (38.1, 59.5)	212327	3044336	2.3 (2.1, 2.6)	3.0 (2.4, 4.0)
Burkina Faso	Est Region	Sep-2016	Phase 1	-	-	-	11384	36.7 (26.2, 47.5)*	30977	307976	3.0 (2.0, 4.3)	3.4 (2.1, 5.5)
Burkina Faso	Est Region	Sep-2017	Phase 1	-	-	-	11650	43.5 (33.9, 53.1)	26774	314967	2.3 (1.7, 3.0)	3.7 (2.7, 5.4)
Burkina Faso	Hauts Bassins Region	Sep-2016	Phase 1	-	-	-	7206	36.7 (26.2, 47.5)*	19608	293835	0.9 (0.4, 1.9)	7.8 (3.3, 17.6)
Burkina Faso	Hauts Bassins Region	Sep-2017	Phase 1	-	-	-	6973	36.7 (26.2, 47.5)*	18974	300234	2.1 (1.3, 3.2)	3.1 (1.8, 5.5)
Burkina Faso	Nord Region	Sep-2016	Phase 1	-	-	-	13197	36.7 (26.2, 47.5)*	35911	271931	2.1 (1.3, 3.3)	6.4 (3.7, 11.8)
Burkina Faso	Nord Region	Sep-2017	Phase 1	-	-	-	11838	36.7 (26.2, 47.5)*	32213	276892	1.7 (1.0, 2.8)	6.8 (3.8, 12.5)
Burkina Faso	Plateau Central Region	Sep-2016	Phase 1	-	-	-	4203	36.7 (26.2, 47.5)*	11437	155695	1.3 (0.6, 2.6)	5.8 (2.6, 13.7)
Burkina Faso	Plateau Central Region	Sep-2017	Phase 1	-	-	-	3598	36.7 (26.2, 47.5)*	9791	158430	1.3 (0.8, 2.2)	4.6 (2.6, 8.6)
Burkina Faso	Sahel Region	Sep-2016	Phase 1	-	-	-	13356	36.7 (26.2, 47.5)*	36343	224193	2.1 (1.2, 3.6)	7.9 (4.2, 14.8)
Burkina Faso	Sahel Region	Sep-2017	Phase 1	-	-	-	12230	36.7 (26.2, 47.5)*	33279	228938	4.5 (3.5, 5.8)	3.2 (2.2, 4.9)
Burkina Faso	Sud-Ouest Region	Sep-2016	Phase 1	-	-	-	4878	36.7 (26.2, 47.5)*	13274	139045	2.7 (1.5, 5.0)	3.5 (1.8, 6.9)
Burkina Faso	Sud-Ouest Region	Sep-2017	Phase 1	-	-	-	4987	36.7 (26.2, 47.5)*	13570	141686	1.6 (0.7, 3.8)	6.0 (2.4, 15.2)
Burundi	Gashoho District	Jul-2013	Phase 2	-	-	-	1012	42.5 (35.4, 49.4)*	2381	13218	0.6 (0.1, 2.4)	30.1 (7.0, 106.4)
Chad	Région de Bahr el Ghazel	Jan-2014	Phase 2	No	-	-	12678	41.5 (27.9, 55.2)	30549	54576	2.6 (1.5, 4.4)	21.9 (11.7, 44.2)
Chad	Région de Barh El Gazal	Aug-2016	Phase 3	Yes	-	-	33038	37.0 (26.7, 48.1)*	89243	57475	2.7 (1.8, 4.1)	57.5 (34.2, 98.0)
Chad	Région de Batha	Jan-2014	Phase 2	No	-	-	12621	19.7 (10.9, 28.5)	64095	111353	5.3 (3.5, 7.8)	10.9 (6.3, 21.6)

Country	Sub-national location	Date	IPC Phase	Prevalence estimate taken during hungry season	MAM treatment available	Acute emergency	SAM treatment admissions (n)	Coverage estimate (%); [*denotes country average]	Estimated incident cases (n)	Population (n)	Prevalence (%)	K estimate
Chad	Région de Batha	Aug-2016	Phase 3	Yes	-	-	47821	37.0 (26.7, 48.1)*	129176	117269	5.4 (3.8, 7.7)	20.3 (13.1, 33.1)
Chad	Région de Guera	Jan-2014	Phase 1	No	-	-	12069	36.3 (26.6, 45.9)	33271	116936	1.9 (1.0, 3.4)	15.3 (7.6, 29.6)
Chad	Région de Guéra	Aug-2016	Phase 2	Yes	-	-	43119	37.0 (26.7, 48.1)*	116474	123148	4.0 (2.4, 6.5)	23.6 (13.5, 42.3)
Chad	Région de Hadjer Lamis	Jan-2014	Phase 1	No	-	-	9028	40.3 (30.3, 50.3)	22405	118409	2.7 (1.6, 4.5)	7.1 (3.9, 12.5)
Chad	Région de Hadjer Lamis	Aug-2016	Phase 1	Yes	-	-	29741	37.0 (26.7, 48.1)*	80337	124700	4.1 (2.9, 5.8)	15.5 (10.0, 24.9)
Chad	Région de Kanem	Jan-2014	Phase 2	No	-	-	23891	36.3 (26.9, 47.2)	65725	74942	1.8 (1.0, 3.2)	49.8 (25.6, 99.3)
Chad	Région de Kanem	Aug-2016	Phase 3	Yes	-	-	77776	37.0 (26.7, 48.1)*	210091	78923	1.8 (0.8, 3.7)	149.4 (66.9, 351.4)
Chad	Région de Lac	Jan-2014	Phase 1	No	-	-	12651	37.0 (26.7, 48.1)*	34173	95353	2.4 (1.4, 3.9)	15.2 (8.3, 26.7)
Chad	Région de Lac	Aug-2016	Phase 3	Yes	-	-	55407	37.0 (26.7, 48.1)*	149667	100418	8.0 (5.5, 11.5)	18.7 (12.0, 29.2)
Chad	Région de Logone occidental	Aug-2016	Phase 1	Yes	-	-	2892	37.0 (26.7, 48.1)*	7812	152098	5.6 (4.1, 7.7)	0.9 (0.6, 1.5)
Chad	Région de Logone orientale	Aug-2016	Phase 1	Yes	-	-	7340	37.0 (26.7, 48.1)*	19827	172563	2.1 (1.3, 3.5)	5.4 (3.0, 10.1)
Chad	Région de Mandoul	Aug-2016	Phase 1	Yes	-	-	2877	37.0 (26.7, 48.1)*	7771	141720	4.9 (3.5, 6.7)	1.1 (0.7, 1.8)
Chad	Région de Moyen Chari	Aug-2016	Phase 1	Yes	-	-	1141	37.0 (26.7, 48.1)*	3082	129943	3.5 (2.4, 5.1)	0.7 (0.4, 1.2)
Chad	Région de N'Djamena	Jan-2014	Phase 1	No	-	-	16820	37.0 (26.7, 48.1)*	45435	208573	0.9 (0.4, 2.2)	23.3 (9.4, 54.9)
Chad	Région de Ndjamena	Aug-2016	Phase 1	Yes	-	-	62231	37.0 (26.7, 48.1)*	168100	219653	3.1 (2.3, 4.2)	24.8 (16.0, 37.9)
Chad	Région de Ouaddai	Jan-2014	Phase 1	No	-	-	17077	37.0 (26.7, 48.1)*	46129	135285	1.6 (0.8, 3.4)	20.8 (9.6, 48.1)
Chad	Région de Ouaddaï	Aug-2016	Phase 2	Yes	-	-	54662	37.0 (26.7, 48.1)*	147655	142472	3.5 (2.5, 4.9)	29.5 (19.3, 46.7)
Chad	Région de Salamat	Jan-2014	Phase 1	No	-	-	8876	43.1 (32.6, 53.6)	20610	64759	3.7 (2.7, 5.1)	8.6 (5.6, 13.4)
Chad	Région de Salamat	Aug-2016	Phase 1	Yes	-	-	29668	37.0 (26.7, 48.1)*	80140	68199	3.0 (2.1, 4.2)	39.6 (25.8, 64.2)
Chad	Région de Sila	Jan-2014	Phase 1	No	-	-	8948	40.9 (29.9, 51.9)	21859	53470	4.1 (2.8, 6.1)	9.9 (6.3, 16.8)
Chad	Région de Sila	Aug-2016	Phase 3	Yes	-	-	31390	37.0 (26.7, 48.1)*	84792	56310	2.6 (1.8, 3.7)	57.8 (35.7, 90.0)
Chad	Région de Wadi Fira	Jan-2014	Phase 2	No	-	-	10070	36.4 (26.9, 46.0)	27627	80831	1.5 (0.8, 2.8)	22.9 (11.7, 48.4)

Country	Sub-national location	Date	IPC Phase	Prevalence estimate taken during hungry season	MAM treatment available	Acute emergency	SAM treatment admissions (n)	Coverage estimate (%); [*denotes country average]	Estimated incident cases (n)	Population (n)	Prevalence (%)	K estimate
Chad	Région de Wadi Fira	Aug-2016	Phase 3	Yes	-	-	36296	37.0 (26.7, 48.1)*	98044	85125	4.4 (3.1, 6.2)	26.1 (16.6, 42.6)
DRC	Kalomba health zone	Oct-2015	-	Yes	No	No	2262	37.9 (28.2, 47.5)	5973	25434	2.3 (1.3, 4.0)	10.2 (5.4, 19.3)
DRC	Banyakiri health zone	Jan-2016	-	No	-	-	1514	34.2 (23.9, 44.6)*	4427	37444	5.4 (4.1, 7.2)	2.2 (1.4, 3.4)
DRC	Banyakiri health zone	Sep-2017	Phase 1	Yes	-	-	1985	34.2 (23.9, 44.6)*	5804	41737	2.2 (1.4, 3.6)	6.2 (3.6, 11.6)
Ethiopia	Abaala Woreda	May-2015	Phase 2	Yes	Yes	No	478	48.8 (37.4, 60.4)*	979	7072	2.6 (1.6, 4.2)	5.4 (3.2, 9.6)
Ethiopia	Abaala Woreda	Dec-2016	Phase 2	Yes	Yes	No	269	48.8 (37.4, 60.4)*	551	7143	0.7 (0.3, 2.0)	10.4 (4.0, 28.6)
Ethiopia	Adaar Woreda	May-2015	Phase 2	Yes	Yes	Yes	1422	48.8 (37.4, 60.4)*	2913	8811	2.5 (1.5, 4.3)	13.1 (7.3, 24.0)
Ethiopia	Adaar Woreda	Oct-2016	Phase 2	No	Yes	Yes	627	48.8 (37.4, 60.4)*	1284	8899	1.3 (0.6, 2.9)	11.2 (4.9, 25.8)
Ethiopia	Bona Zuria Woreda	May-2015	Phase 1	Yes	No	No	1109	48.8 (37.4, 60.4)*	2272	20761	3.3 (2.1, 5.2)	3.3 (2.0, 5.7)
Ethiopia	Bona Zuria Woreda	Oct-2016	Phase 1	No	No	No	501	48.8 (37.4, 60.4)*	1026	20969	1.1 (0.5, 2.3)	4.6 (2.0, 10.8)
Ethiopia	Bulehora Woreda	Mar-2014	Phase 2	No	No	No	1422	48.8 (37.4, 60.4)*	2913	55796	1.0 (0.4, 2.3)	5.5 (2.2, 13.7)
Ethiopia	Dessie Zuria Woreda	May-2015	Phase 2	No	Yes	No	525	48.8 (37.4, 60.4)*	1075	21903	1.4 (0.6, 3.1)	3.5 (1.5, 8.1)
Ethiopia	Gubalafto Woreda	May-2015	Phase 2	No	Yes	Yes	409	48.8 (37.4, 60.4)*	838	16821	1.4 (0.6, 3.3)	3.6 (1.4, 8.9)
Ethiopia	Hadelela Woreda	May-2015	Phase 2	Yes	Yes	No	1128	48.8 (37.4, 60.4)*	2311	5663	2.6 (1.4, 4.7)	15.6 (8.5, 30.2)
Ethiopia	Hadelela Woreda	Nov-2016	Phase 2	Yes	Yes	Yes	1217	48.8 (37.4, 60.4)*	2493	5720	1.9 (0.9, 3.9)	23.3 (10.6, 50.9)
Ethiopia	Halaba Woreda	May-2015	Phase 3	Yes	Yes	Yes	2258	48.8 (37.4, 60.4)*	4625	42880	1.1 (0.5, 2.3)	9.6 (4.5, 22.0)
Ethiopia	Konso Woreda	May-2015	Phase 1	Yes	Yes	No	1592	48.8 (37.4, 60.4)*	3261	36967	1.4 (0.7, 2.8)	6.3 (2.9, 12.8)
Ethiopia	Meiso Woreda	May-2015	Phase 3	No	Yes	Yes	2878	48.8 (37.4, 60.4)*	5895	25284	2.1 (1.2, 3.5)	11.2 (6.5, 20.3)
Ethiopia	Meiso Woreda	Aug-2016	Phase 3	Yes	Yes	Yes	1118	50.9 (37.8, 63.7)	2196	25538	2.2 (1.4, 3.6)	3.9 (2.3, 7.0)
Ethiopia	Midhega Tola Woreda	May-2015	Phase 3	No	Yes	Yes	1428	48.8 (37.4, 60.4)*	2925	14319	2.4 (1.4, 4.2)	8.4 (4.7, 15.7)
Ethiopia	Midhega Tola Woreda	Aug-2016	Phase 3	Yes	Yes	Yes	951	48.8 (37.4, 60.4)*	1948	14463	2.0 (1.1, 3.6)	6.7 (3.7, 12.9)
Ethiopia	Miyo Woreda	May-2015	Phase 3	No	No	Yes	203	48.8 (37.4, 60.4)*	416	9751	0.2 (0.0, 1.6)	18.9 (2.4, 93.5)
Ethiopia	Miyo Woreda	Aug-2016	Phase 2	Yes	No	Yes	251	48.8 (37.4, 60.4)*	514	9849	0.4 (0.1, 1.7)	12.0 (2.7, 43.4)
Ethiopia	Raya Azebo Woreda	May-2015	Phase 2	No	Yes	No	275	48.8 (37.4, 60.4)*	563	20147	0.7 (0.2, 1.8)	4.2 (1.5, 10.3)

Country	Sub-national location	Date	IPC Phase	Prevalence estimate taken during hungry season	MAM treatment available	Acute emergency	SAM treatment admissions (n)	Coverage estimate (%); [*denotes country average]	Estimated incident cases (n)	Population (n)	Prevalence (%)	K estimate
Ethiopia	Raya Azebo Woreda	Jul-2016	Phase 3	Yes	Yes	No	283	48.8 (37.4, 60.4)*	580	20349	1.3 (0.6, 2.5)	2.2 (1.1, 4.4)
Ethiopia	Saesi Tsaeda Emba Woreda	May-2015	Phase 2	No	Yes	Yes	405	48.8 (37.4, 60.4)*	830	20788	1.5 (0.8, 2.9)	2.6 (1.3, 5.4)
Ethiopia	Saesi Tsaeda Emba Woreda	Jul-2016	Phase 3	Yes	Yes	Yes	307	48.8 (37.4, 60.4)*	629	20997	1.1 (0.5, 2.4)	2.7 (1.2, 6.5)
Ethiopia	Sekota Zuria Woreda	May-2015	Phase 3	No	Yes	Yes	1962	42.2 (30.0, 54.3)	4652	19466	3.0 (1.9, 4.7)	7.9 (4.7, 13.6)
Ethiopia	Siraro Woreda	May-2015	Phase 3	No	Yes	Yes	3516	48.8 (37.4, 60.4)*	7202	26916	1.8 (1.0, 3.2)	15.0 (7.8, 28.5)
Ethiopia	Siraro Woreda	Aug-2016	Phase 3	Yes	Yes	Yes	2703	48.8 (37.4, 60.4)*	5537	27186	1.7 (0.9, 3.1)	12.3 (6.7, 24.1)
Ethiopia	Tankua Abergele Woreda	May-2015	Phase 3	No	Yes	Yes	329	48.8 (37.4, 60.4)*	674	13573	1.0 (0.5, 2.0)	4.7 (2.3, 9.9)
Ethiopia	Tankua Abergele Woreda	Jul-2016	Phase 3	Yes	Yes	Yes	398	48.8 (37.4, 60.4)*	815	13709	1.2 (0.6, 2.3)	4.9 (2.5, 9.9)
Ethiopia	Wenago Woreda	May-2015	Phase 1	Yes	No	No	1613	48.8 (37.4, 60.4)*	3304	24371	1.1 (0.5, 2.4)	12.6 (5.7, 32.5)
Ghana	Northern Region	Dec-2013	-	No	-	-	3574	5.4 (0.2, 26.2)	65578	406148	2.8 (2.0, 3.9)	5.8 (0.8, 30.8)
Ghana	Upper East Region	Dec-2013	-	No	-	-	1472	25.9 (11.6, 50.2)	5694	139516	2.7 (1.8, 4.1)	1.5 (0.7, 3.4)
Ghana	Upper West Region	Dec-2013	-	No	-	-	916	7.7 (0.2, 36.2)	11896	94036	3.1 (2.0, 4.7)	4.1 (0.5, 24.0)
Kenya	Garissa County	May-2017	Phase 3	No	-	-	2607	48.8 (36.9, 60.8)*	5340	169560	1.9 (1.1, 3.1)	1.7 (1.0, 2.9)
Kenya	Isiolo County	May-2012	Phase 2	No	-	No	332	42.0 (26.0, 58.0)	790	19571	3.7 (2.5, 5.4)	1.1 (0.7, 2.0)
Kenya	Isiolo County	Feb-2018	Phase 3	Yes	-	-	678	48.8 (36.9, 60.8)*	1389	23735	3.5 (2.3, 5.2)	1.7 (1.0, 2.9)
Kenya	Kajiado County	Jan-2018	Phase 3	-	-	-	2117	48.8 (36.9, 60.8)*	4337	130965	3.1 (2.0, 4.8)	1.1 (0.7, 1.8)
Kenya	Kitui District	Apr-2011	Phase 2	No	-	Yes	1022	48.8 (36.9, 60.8)*	2094	135601	1.2 (0.6, 2.3)	1.3 (0.6, 3.0)
Kenya	Laikipia District	Aug-2012	Phase 2	No	-	No	404	41.9 (30.9, 52.9)	964	69916	4.7 (3.1, 6.9)	0.3 (0.2, 0.5)
Kenya	Laisamis Subcounty	May-2017	Phase 3	No	-	-	762	54.6 (41.9, 67.3)	1395	8798	7.1 (5.0, 10.0)	2.2 (1.5, 3.5)
Kenya	Makueni District	Apr-2011	Phase 2	No	-	Yes	1152	48.8 (36.9, 60.8)*	2360	118438	0.3 (0.1, 1.4)	5.7 (1.4, 19.0)
Kenya	Makueni District	Mar-2012	Phase 2	No	-	No	405	48.8 (36.9, 60.8)*	830	120806	0.4 (0.1, 1.6)	1.7 (0.4, 6.4)
Kenya	Mandera County	May-2017	Phase 3	No	-	-	7720	48.8 (36.9, 60.8)*	15814	279151	6.0 (4.6, 7.9)	0.9 (0.7, 1.4)
Kenya	Moyale Subcounty	May-2017	Phase 3	No	-	-	841	48.8 (36.9, 60.8)*	1723	11594	1.4 (0.6, 3.3)	10.6 (4.2, 26.9)
Kenya	Mwingi District	Oct-2009	Phase 4	-	-	No	1484	48.8 (36.9, 60.8)*	3040	45439	2.0 (1.2, 3.3)	3.4 (1.9, 5.7)

Country	Sub-national location	Date	IPC Phase	Prevalence estimate taken during hungry season	MAM treatment available	Acute emergency	SAM treatment admissions (n)	Coverage estimate (%); [*denotes country average]	Estimated incident cases (n)	Population (n)	Prevalence (%)	K estimate
Kenya	Mwingi District	Apr-2011	Phase 2	-	-	Yes	1010	48.8 (36.9, 60.8)*	2069	48607	1.7 (0.9, 3.1)	2.5 (1.4, 4.8)
Kenya	Narok County	Jan-2018	Phase 2	-	-	-	869	48.8 (36.9, 60.8)*	1780	162140	2.1 (1.2, 3.7)	0.5 (0.3, 0.9)
Kenya	North Horr Subcounty	May-2017	Phase 3	-	-	-	785	48.8 (36.9, 60.8)*	1608	9501	6.9 (4.8, 9.8)	2.5 (1.6, 3.9)
Kenya	Saku Subcounty	May-2017	Phase 3	-	-	-	350	53.0 (38.4, 67.6)	661	8069	1.6 (0.6, 4.1)	5.2 (1.9, 13.7)
Kenya	Samburu County	Jun-2017	Phase 3	-	-	-	905	48.8 (36.9, 60.8)*	1854	41190	4.4 (2.9, 6.5)	1.0 (0.6, 1.6)
Kenya	Turkana Central Subcounty	Feb-2017	Phase 3	-	-	-	2921	48.8 (36.9, 60.8)*	5984	15308	8.2 (6.5, 10.3)	4.7 (3.4, 6.7)
Kenya	Turkana Central Subcounty	Jun-2017	Phase 3	-	-	-	2695	48.8 (36.9, 60.8)*	5521	15308	9.5 (7.4, 11.9)	3.8 (2.7, 5.5)
Kenya	Turkana North Subcounty	Feb-2017	Phase 3	-	-	-	1253	48.8 (36.9, 60.8)*	2567	15479	9.4 (7.4, 11.7)	1.8 (1.3, 2.5)
Kenya	Turkana North Subcounty	Jun-2017	Phase 3	-	-	-	834	48.8 (36.9, 60.8)*	1708	15479	9.6 (7.6, 12.2)	1.1 (0.8, 1.6)
Kenya	Turkana South Subcounty	Feb-2017	Phase 3	-	-	-	2018	48.8 (36.9, 60.8)*	4134	9984	7.2 (5.1, 10.1)	5.7 (3.8, 8.8)
Kenya	Turkana South Subcounty	Jun-2017	Phase 3	-	-	-	1876	48.8 (36.9, 60.8)*	3843	9984	13.2 (10.6, 16.2)	2.9 (2.2, 4.2)
Kenya	Turkana West Subcounty	Feb-2017	Phase 3	-	-	-	8347	48.8 (36.9, 60.8)*	17098	30960	4.7 (3.1, 6.9)	11.8 (7.5, 20.0)
Kenya	Turkana West Subcounty	Jun-2017	Phase 3	-	-	-	7846	48.8 (36.9, 60.8)*	16072	30960	8.6 (6.5, 11.4)	6.0 (4.2, 8.9)
Kenya	West Pokot County	Mar-2013	Phase 2	Yes	-	No	2620	21.7 (12.7, 30.7)	12074	86110	1.3 (0.6, 2.7)	10.7 (4.9, 27.0)
Kenya	West Pokot District	May-2012	Phase 2	No	-	No	1038	33.5 (23.0, 44.0)	3098	83117	2.5 (1.4, 4.6)	1.5 (0.8, 3.1)
Liberia	Bomi County	May-2016	Phase 1	No	No	No	623	24.8 (15.6, 37.0)*	2512	15906	2.4 (1.3, 4.6)	6.5 (3.0, 14.2)
Liberia	Bong County	May-2016	Phase 1	No	No	No	1593	24.8 (15.6, 37.0)*	6423	63055	1.7 (0.8, 3.6)	6.0 (2.6, 14.9)
Liberia	Gbarpolu County	May-2016	Phase 1	No	No	No	376	24.8 (15.6, 37.0)*	1516	25703	1.9 (1.0, 3.8)	3.1 (1.4, 7.1)
Liberia	Grand Bassa County	May-2016	Phase 1	No	No	No	869	24.8 (15.6, 37.0)*	3504	211439	3.0 (1.5, 5.6)	0.6 (0.3, 1.2)

Country	Sub-national location	Date	IPC Phase	Prevalence estimate taken during hungry season	MAM treatment available	Acute emergency	SAM treatment admissions (n)	Coverage estimate (%); [*denotes country average]	Estimated incident cases (n)	Population (n)	Prevalence (%)	K estimate
Liberia	Grand Cape Mount County	May-2016	Phase 1	No	No	No	591	24.8 (15.6, 37.0)*	2383	15767	2.9 (1.6, 5.1)	5.2 (2.4, 11.0)
Liberia	Grand Gedeh County	May-2016	Phase 1	No	No	No	577	24.8 (15.6, 37.0)*	2327	10950	0.4 (0.1, 3.1)	48.5 (5.3, 317.4)
Liberia	Grand Kru County	May-2016	Phase 1	No	No	No	358	24.8 (15.6, 37.0)*	1444	87360	1.9 (0.9, 4.2)	0.9 (0.4, 2.2)
Liberia	Lofa County	May-2016	Phase 1	No	No	No	2073	24.8 (15.6, 37.0)*	8359	41918	1.4 (0.5, 3.6)	14.7 (4.7, 43.6)
Liberia	Margibi County	May-2016	Phase 1	No	No	Yes	827	24.8 (15.6, 37.0)*	3335	23684	3.4 (1.6, 7.0)	4.2 (1.8, 9.7)
Liberia	Maryland County	May-2016	Phase 1	No	No	No	942	24.8 (15.6, 37.0)*	3798	52349	1.5 (0.6, 4.0)	4.8 (1.7, 14.2)
Liberia	Montserrado County	May-2016	Phase 1	No	No	No	6023	24.8 (15.6, 37.0)*	24286	39693	1.9 (0.8, 4.2)	32.4 (13.1, 76.3)
Liberia	Nimba County	May-2016	Phase 1	No	No	No	1016	24.8 (15.6, 37.0)*	4097	13521	1.1 (0.4, 3.0)	26.4 (8.8, 72.1)
Liberia	River Gee County	May-2016	Phase 1	No	No	No	316	24.8 (15.6, 37.0)*	1274	19396	0.4 (0.1, 3.1)	14.8 (1.7, 62.0)
Liberia	Rivercess County	May-2016	Phase 1	No	No	No	459	24.8 (15.6, 37.0)*	1851	12629	1.6 (0.6, 4.2)	9.3 (3.0, 24.9)
Liberia	Sinoe County	May-2016	Phase 1	No	No	No	375	24.8 (15.6, 37.0)*	1512	24027	0.8 (0.2, 3.2)	7.7 (1.7, 28.2)
Madagascar	District d'Amboasary	Mar-2017	Phase 3	Yes	Yes	Yes	2930	28.7 (20.9, 37.6)*	10209	52665	2.5 (1.6, 3.9)	7.6 (4.6, 13.0)
Madagascar	District d'Ambovombe	Apr-2017	Phase 2	No	Yes	Yes	3929	28.7 (20.9, 37.6)*	13690	53334	2.4 (1.6, 3.7)	10.6 (6.5, 17.7)
Madagascar	District d'Ampanihy	Apr-2017	Phase 3	No	Yes	Yes	3270	28.7 (20.9, 37.6)*	11394	62357	1.7 (1.1, 2.5)	10.8 (6.5, 17.9)
Madagascar	District de Bekily	Apr-2017	Phase 2	No	Yes	Yes	1794	28.7 (20.9, 37.6)*	6251	33059	2.3 (1.5, 3.6)	8.1 (4.8, 13.8)
Madagascar	District de Beloha	Feb-2017	Phase 3	Yes	Yes	Yes	1665	28.7 (20.9, 37.6)*	5801	21991	3.8 (2.6, 5.3)	7.0 (4.6, 11.0)
Madagascar	District de Betioky	May-2017	Phase 3	No	Yes	Yes	1259	28.7 (20.9, 37.6)	4387	46504	1.9 (1.2, 3.0)	4.9 (2.8, 8.3)
Madagascar	District de Taolagnaro	Mar-2017	Phase 3	Yes	No	Yes	1608	28.7 (20.9, 37.6)*	5603	62434	1.2 (0.6, 2.5)	7.6 (3.4, 15.8)
Madagascar	District de Tsihombe	Mar-2017	Phase 3	Yes	Yes	Yes	2296	28.7 (20.9, 37.6)*	8000	22992	2.2 (1.4, 3.4)	15.8 (9.3, 28.6)
Mali	Région de Bamako	Jul-2014	Phase 1	Yes	-	-	5721	34.9 (25.7, 44.4)*	16375	357300	3.8 (2.6, 5.6)	1.2 (0.7, 1.9)
Mali	Région de Bamako	May-2015	Phase 1	No	-	-	6971	34.9 (25.7, 44.4)*	19953	364500	1.8 (1.0, 3.0)	3.1 (1.7, 6.0)
Mali	Région de Bamako	Jul-2016	Phase 1	Yes	-	-	5183	34.9 (25.7, 44.4)*	14835	372600	2.5 (1.7, 3.7)	1.6 (1.0, 2.5)
Mali	Région de Bamako	Aug-2017	Phase 1	Yes	-	-	4073	34.9 (25.7, 44.4)*	11658	380700	2.7 (1.9, 3.7)	1.1 (0.7, 1.8)
Mali	Région de Gao	May-2015	Phase 2	No	-	-	10065	34.9 (25.7, 44.4)*	28809	108900	2.5 (1.8, 3.7)	10.4 (6.7, 16.9)
Mali	Région de Gao	Jul-2016	Phase 2	Yes	-	-	9700	34.9 (25.7, 44.4)*	27764	111600	3.8 (2.7, 5.2)	6.6 (4.4, 10.0)

Country	Sub-national location	Date	IPC Phase	Prevalence estimate taken during hungry season	MAM treatment available	Acute emergency	SAM treatment admissions (n)	Coverage estimate (%); [*denotes country average]	Estimated incident cases (n)	Population (n)	Prevalence (%)	K estimate
Mali	Région de Gao	Aug-2017	Phase 2	Yes	-	-	9441	34.9 (25.7, 44.4)*	27023	114300	3.2 (2.1, 5.0)	7.4 (4.4, 12.7)
Mali	Région de Kayes	Jul-2014	Phase 2	Yes	-	-	19143	34.9 (25.0, 45.1)	54900	393300	2.1 (1.2, 3.5)	6.7 (3.6, 12.5)
Mali	Région de Kayes	May-2015	Phase 1	No	-	-	19222	32.0 (21.4, 42.9)	60156	401400	3.7 (2.7, 5.1)	4.0 (2.6, 6.6)
Mali	Région de Kayes	Jul-2016	Phase 1	Yes	-	-	16642	34.9 (25.7, 44.4)*	47634	410400	1.6 (0.9, 2.7)	7.4 (3.9, 14.1)
Mali	Région de Kayes	Aug-2017	Phase 2	Yes	-	-	14646	34.9 (25.7, 44.4)*	41921	419400	4.0 (3.1, 5.2)	2.5 (1.8, 3.7)
Mali	Région de Kidal	Aug-2017	Phase 1	Yes	-	-	481	34.9 (25.7, 44.4)*	1377	14400	1.2 (0.5, 3.2)	8.0 (2.9, 22.2)
Mali	Région de Koulikoro	Jul-2014	Phase 2	Yes	-	-	24696	29.3 (22.7, 36.0)	84203	477900	3.2 (2.1, 4.8)	5.5 (3.4, 8.9)
Mali	Région de Koulikoro	May-2015	Phase 1	No	-	-	26685	44.4 (33.9, 55.0)	60064	480600	2.8 (1.9, 4.3)	4.4 (2.7, 7.3)
Mali	Région de Koulikoro	Jul-2016	Phase 1	Yes	-	-	18832	34.9 (25.7, 44.4)*	53902	498600	2.1 (1.4, 3.1)	5.2 (3.2, 8.5)
Mali	Région de Koulikoro	Aug-2017	Phase 2	Yes	-	-	16415	34.9 (25.7, 44.4)*	46984	509400	2.8 (2.0, 3.9)	3.3 (2.2, 5.2)
Mali	Région de Mopti	Jul-2014	Phase 2	Yes	-	-	19035	29.5 (21.6, 37.7)	64423	401400	3.0 (2.6, 3.4)	5.4 (4.1, 7.7)
Mali	Région de Mopti	May-2015	Phase 1	No	-	-	27779	34.9 (25.7, 44.4)*	79511	409500	2.0 (1.3, 3.2)	9.6 (5.8, 16.9)
Mali	Région de Mopti	Jul-2016	Phase 1	Yes	-	-	24517	34.9 (25.7, 44.4)*	70174	419400	2.8 (1.9, 4.2)	6.0 (3.6, 10.2)
Mali	Région de Mopti	Aug-2017	Phase 2	Yes	-	-	24582	38.5 (28.5, 48.4)	63913	428400	1.8 (1.2, 2.8)	8.2 (4.9, 13.3)
Mali	Région de Ségou	Jul-2014	Phase 1	Yes	-	-	24811	41.9 (31.5, 52.3)	59196	460800	3.1 (2.1, 4.6)	4.1 (2.6, 6.6)
Mali	Région de Ségou	May-2015	Phase 1	No	-	-	26573	36.5 (25.9, 47.1)	72846	470700	1.6 (0.9, 2.8)	9.8 (5.1, 18.1)
Mali	Région de Ségou	Jul-2016	Phase 1	Yes	-	-	21820	34.9 (25.7, 44.4)*	62454	481500	5.0 (3.8, 6.6)	2.6 (1.8, 3.9)
Mali	Région de Ségou	Aug-2017	Phase 1	Yes	-	-	20701	34.9 (25.7, 44.4)*	59252	492300	3.6 (2.6, 5.1)	3.3 (2.1, 5.2)
Mali	Région de Sikasso	Jul-2014	Phase 1	Yes	-	-	20133	28.1 (20.2, 36.0)	71701	521100	2.6 (2.3, 3.1)	5.2 (3.9, 7.6)
Mali	Région de Sikasso	May-2015	Phase 1	No	-	-	20326	34.9 (25.7, 44.4)*	58178	531900	3.6 (2.5, 5.0)	3.1 (2.0, 4.7)
Mali	Région de Sikasso	Jul-2016	Phase 1	Yes	-	-	15770	34.9 (25.7, 44.4)*	45138	544500	1.9 (1.2, 3.3)	4.3 (2.4, 7.5)
Mali	Région de Sikasso	Aug-2017	Phase 1	Yes	-	-	13524	34.9 (25.7, 44.4)*	38709	556200	2.6 (2.0, 3.4)	2.7 (1.9, 4.0)
Mali	Région de Tombouctou	Jul-2014	Phase 2	Yes	-	-	9157	18.8 (13.1, 22.8)	48707	133200	3.4 (2.2, 5.2)	10.9 (6.8, 19.9)
Mali	Région de Tombouctou	May-2015	Phase 2	No	-	-	14892	34.9 (25.7, 44.4)*	42625	135900	4.4 (3.3, 5.9)	7.1 (4.8, 10.8)

Country	Sub-national location	Date	IPC Phase	Prevalence estimate taken during hungry season	MAM treatment available	Acute emergency	SAM treatment admissions (n)	Coverage estimate (%); [*denotes country average]	Estimated incident cases (n)	Population (n)	Prevalence (%)	K estimate
Mali	Région de Tombouctou	Jul-2016	Phase 2	Yes	-	-	12985	34.9 (25.7, 44.4)*	37166	138600	4.4 (3.2, 6.1)	6.0 (4.0, 9.2)
Mali	Région de Tombouctou	Aug-2017	Phase 2	Yes	-	-	11391	34.9 (25.7, 44.4)*	32604	142200	2.9 (1.8, 4.6)	7.9 (4.7, 13.1)
Mauritania	Gorgol Wilaya	Jun-2012	-	Yes	-	-	2892	32.1 (19.9, 46.3)	9009	59914	1.2 (0.6, 2.6)	12.1 (5.3, 30.5)
Mauritania	Hodh el Gharbi Wilaya	Jun-2012	-	Yes	-	-	911	33.1 (20.8, 48.0)*	2748	51548	2.4 (1.5, 3.9)	2.2 (1.2, 4.4)
Myanmar	Buthidung Township	Sep-2015	-	Yes	-	Yes	4996	56.7 (48.3, 64.7)	8811	48505	2.9 (1.7, 4.7)	6.3 (3.7, 10.4)
Myanmar	Maungdaw Township	Sep-2015	-	Yes	-	Yes	6581	29.7 (21.4, 37.9)	22158	84302	5.4 (3.9, 7.5)	4.9 (3.2, 7.8)
Myanmar	Pauktaw IDP camp	Jul-2016	-	Yes	-	Yes	422	82.1 (71.6, 92.7)	514	3946	1.5 (0.8, 2.8)	8.7 (4.5, 17.7)
Myanmar	Sittwe Rural IDP	Jul-2016	-	Yes	-	Yes	2100	65.3 (53.8, 76.7)	3216	9741	0.9 (0.3, 2.4)	36.5 (13.1, 100.1)
Myanmar	Sittwe Urban IDP	Jul-2016	-	Yes	-	Yes	110	61.1 (51.9, 70.2)*	180	390	0.7 (0.3, 2.0)	60.0 (22.4, 174.8)
Niger	Agadez Region	Aug-2015	Phase 1	Yes	Yes	-	7602	43.9 (35.2, 52.6)	17335	82411	2.9 (1.5, 5.6)	7.2 (3.6, 13.6)
Niger	Diffa Region	Aug-2015	Phase 3	Yes	Yes	-	16577	39.5 (30.3, 48.6)*	41974	107355	3.8 (2.3, 6.1)	10.4 (6.3, 18.4)
Niger	Dosso Region	Aug-2015	Phase 1	Yes	Yes	-	31211	39.5 (30.3, 48.6)*	79029	90830	6.1 (4.1, 8.9)	14.3 (9.2, 22.4)
Niger	Maradi Region	Aug-2015	Phase 1	Yes	Yes	-	97523	39.5 (30.3, 48.6)*	246937	737693	7.0 (5.2, 9.5)	4.8 (3.3, 7.0)
Niger	Niamey Region	Aug-2015	Phase 1	Yes	Yes	-	7136	39.5 (30.3, 48.6)*	18069	168128	4.3 (2.5, 7.3)	2.5 (1.4, 4.7)
Niger	Tahoua Region	Aug-2015	Phase 2	Yes	Yes	-	69397	39.5 (30.3, 48.6)*	175719	661278	5.0 (3.1, 7.9)	5.4 (3.3, 9.4)
Niger	Tillabery Region	Aug-2015	Phase 2	Yes	Yes	-	36820	56.3 (45.7, 66.9)	65391	556099	2.8 (1.6, 4.7)	4.2 (2.5, 7.2)
Niger	Zinder Region	Aug-2015	Phase 2	Yes	Yes	-	119326	34.6 (25.2, 41.2)	344584	752585	5.1 (3.4, 7.7)	8.9 (5.9, 15.2)
Niger	Agadez Region	Aug-2016	Phase 2	Yes	Yes	-	6951	39.5 (30.3, 48.6)*	17601	83808	2.5 (1.7, 3.7)	8.4 (5.2, 13.5)
Niger	Diffa Region	Aug-2016	Phase 3	Yes	Yes	-	16731	39.5 (30.3, 48.6)*	42364	106470	2.0 (1.6, 2.5)	19.7 (14.6, 27.9)
Niger	Dosso Region	Aug-2016	Phase 1	Yes	Yes	-	28166	39.5 (30.3, 48.6)*	71319	422546	1.4 (0.7, 2.8)	11.8 (5.8, 25.0)
Niger	Maradi Region	Aug-2016	Phase 1	Yes	Yes	-	108988	39.5 (30.3, 48.6)*	275967	761168	4.3 (2.8, 6.5)	8.5 (5.3, 13.6)
Niger	Niamey Region	Aug-2016	Phase 1	Yes	Yes	-	8701	39.5 (30.3, 48.6)*	22032	175640	0.5 (0.2, 1.3)	25.7 (9.5, 78.2)
Niger	Tahoua Region	Aug-2016	Phase 2	Yes	Yes	-	73739	39.5 (30.3, 48.6)*	186714	672595	1.3 (0.6, 2.7)	22.1 (9.8, 52.4)
Niger	Tillabery Region	Aug-2016	Phase 1	Yes	Yes	-	38738	39.5 (30.3, 48.6)*	98088	570674	2.9 (1.7, 4.7)	6.0 (3.5, 10.3)

Country	Sub-national location	Date	IPC Phase	Prevalence estimate taken during hungry season	MAM treatment available	Acute emergency	SAM treatment admissions (n)	Coverage estimate (%); [*denotes country average]	Estimated incident cases (n)	Population (n)	Prevalence (%)	K estimate
Niger	Zinder Region	Aug-2016	Phase 1	Yes	Yes	-	110378	39.5 (30.3, 48.6)*	279487	776598	4.5 (2.9, 7.0)	8.0 (4.6, 13.0)
Nigeria	Adamawa	Jul-2013	Phase 1	Yes	-	-	5218	35.3 (26.3, 44.3)*	14777	599298	1.4 (0.7, 2.5)	1.8 (0.9, 3.7)
Nigeria	Adamawa	Mar-2014	Phase 2	No	-	-	6488	27.6 (18.3, 36.8)	23549	614220	2.0 (1.0, 3.9)	2.0 (0.9, 4.4)
Nigeria	Adamawa	Jul-2015	Phase 3	Yes	-	-	19301	35.3 (26.3, 44.3)*	54658	629514	2.2 (1.0, 4.8)	4.0 (1.7, 10.0)
Nigeria	Bauchi	Jul-2013	Phase 1	Yes	-	-	3511	35.3 (26.3, 44.3)*	9943	996145	3.8 (2.6, 5.4)	0.3 (0.2, 0.4)
Nigeria	Bauchi	Mar-2014	Phase 1	No	-	-	10683	19.5 (12.7, 26.2)	54917	1020949	5.5 (4.1, 7.3)	1.0 (0.6, 1.7)
Nigeria	Bauchi	Jul-2015	Phase 1	Yes	-	-	17665	35.3 (26.3, 44.3)*	50025	1046370	3.7 (2.5, 5.5)	1.3 (0.8, 2.2)
Nigeria	Borno	Aug-2010	Phase 3	Yes	-	-	1362	35.3 (26.3, 44.3)*	3857	789902	2.6 (1.5, 4.4)	0.2 (0.1, 0.3)
Nigeria	Borno	Dec-2010	Phase 1	No	-	-	1362	35.3 (26.3, 44.3)*	3857	789902	2.2 (1.2, 3.9)	0.2 (0.1, 0.4)
Nigeria	Borno	Feb-2012	Phase 1	No	-	-	8262	35.3 (26.3, 44.3)*	23397	829729	3.9 (2.7, 5.6)	0.7 (0.5, 1.1)
Nigeria	Borno	Oct-2012	Phase 2	No	-	-	8262	35.3 (26.3, 44.3)*	23397	829729	5.9 (4.3, 7.9)	0.5 (0.3, 0.7)
Nigeria	Borno	Oct-2013	Phase 3	No	-	-	7058	35.3 (26.3, 44.3)*	19987	850389	4.5 (3.2, 6.3)	0.5 (0.3, 0.8)
Nigeria	Borno	Mar-2014	Phase 3	No	-	-	15504	35.3 (26.3, 44.3)*	43906	871563	2.7 (1.6, 4.4)	1.9 (1.1, 3.4)
Nigeria	Borno	Jul-2015	Phase 4	Yes	-	-	42514	35.3 (26.3, 44.3)*	120395	893265	4.4 (3.0, 6.5)	3.1 (1.9, 4.9)
Nigeria	Gombe	Jul-2013	Phase 1	Yes	-	-	6346	35.3 (26.3, 44.3)*	17971	492303	1.1 (0.6, 2.1)	3.3 (1.6, 7.1)
Nigeria	Gombe	Mar-2014	Phase 1	No	-	-	6681	27.3 (17.4, 37.2)	24476	504561	3.4 (2.3, 5.1)	1.4 (0.8, 2.5)
Nigeria	Gombe	Jul-2015	Phase 1	Yes	-	-	11407	35.3 (26.3, 44.3)*	32303	517124	4.4 (2.9, 6.7)	1.4 (0.9, 2.3)
Nigeria	Jigawa	Aug-2010	Phase 3	Yes	-	-	10107	35.3 (26.3, 44.3)*	28622	872469	4.5 (3.1, 6.5)	0.7 (0.5, 1.1)
Nigeria	Jigawa	Dec-2010	Phase 2	No	-	-	10107	35.3 (26.3, 44.3)*	28622	872469	7.1 (5.2, 9.5)	0.5 (0.3, 0.7)
Nigeria	Jigawa	Jul-2011	Phase 1	Yes	-	-	23375	35.3 (26.3, 44.3)*	66195	894194	5.1 (3.7, 6.9)	1.5 (1.0, 2.2)
Nigeria	Jigawa	Sep-2012	Phase 2	Yes	-	-	40642	35.3 (26.3, 44.3)*	115094	916459	5.0 (3.7, 6.7)	2.5 (1.7, 3.8)
Nigeria	Jigawa	Aug-2013	Phase 1	Yes	-	-	45412	40.3 (30.7, 49.8)	112796	939279	5.4 (4.0, 7.2)	2.2 (1.5, 3.4)
Nigeria	Jigawa	Mar-2014	Phase 1	No	-	-	51616	48.5 (41.1, 55.8)	106519	962667	5.3 (3.9, 7.3)	2.1 (1.5, 3.0)
Nigeria	Jigawa	Jul-2015	Phase 1	Yes	-	-	70175	35.3 (26.3, 44.3)*	198728	986637	4.1 (2.8, 5.9)	4.9 (3.2, 7.6)
Nigeria	Kano	Jul-2011	Phase 1	Yes	-	-	6479	35.3 (26.3, 44.3)*	18348	1850915	3.2 (2.1, 4.7)	0.3 (0.2, 0.5)
Nigeria	Kano	Feb-2012	Phase 1	No	-	-	24087	35.3 (26.3, 44.3)*	68212	1897003	2.8 (1.9, 4.2)	1.3 (0.8, 2.1)
Nigeria	Kano	Sep-2012	Phase 1	Yes	-	-	24087	35.3 (26.3, 44.3)*	68212	1897003	3.2 (2.2, 4.6)	1.1 (0.7, 1.7)
Nigeria	Kano	Aug-2013	Phase 1	Yes	-	-	36931	35.3 (26.3, 44.3)*	104584	1944238	4.8 (3.4, 6.6)	1.1 (0.7, 1.7)
Nigeria	Kano	Mar-2014	Phase 1	No	-	-	39205	28.0 (17.9, 38.1)	140045	1992650	5.8 (4.3, 7.9)	1.2 (0.8, 2.1)

Country	Sub-national location	Date	IPC Phase	Prevalence estimate taken during hungry season	MAM treatment available	Acute emergency	SAM treatment admissions (n)	Coverage estimate (%); [*denotes country average]	Estimated incident cases (n)	Population (n)	Prevalence (%)	K estimate
Nigeria	Kano	Jul-2015	Phase 1	Yes	-	-	57195	35.3 (26.3, 44.3)*	161970	2042267	4.3 (2.9, 6.4)	1.8 (1.2, 3.0)
Nigeria	Katsina	Aug-2010	Phase 2	Yes	-	-	10968	35.3 (26.3, 44.3)*	31060	1199432	7.0 (5.4, 9.1)	0.4 (0.3, 0.5)
Nigeria	Katsina	Nov-2010	Phase 1	No	-	-	10968	35.3 (26.3, 44.3)*	31060	1199432	5.8 (4.1, 8.3)	0.4 (0.3, 0.7)
Nigeria	Katsina	Aug-2011	Phase 1	Yes	-	-	45048	35.3 (26.3, 44.3)*	127571	1229298	4.5 (3.3, 6.1)	2.3 (1.6, 3.5)
Nigeria	Katsina	Mar-2012	Phase 1	No	-	-	49373	35.3 (26.3, 44.3)*	139819	1259907	1.6 (1.0, 2.7)	6.9 (4.0, 12.4)
Nigeria	Katsina	Sep-2012	Phase 1	Yes	-	-	49373	35.3 (26.3, 44.3)*	139819	1259907	3.5 (2.5, 4.8)	3.2 (2.1, 5.0)
Nigeria	Katsina	Sep-2013	Phase 1	Yes	-	-	54934	53.4 (43.9, 62.9)	102876	1291279	6.7 (5.2, 8.6)	1.2 (0.9, 1.6)
Nigeria	Katsina	Mar-2014	Phase 1	No	-	-	78856	32.4 (22.1, 42.6)	243480	1323432	4.3 (3.0, 6.1)	4.3 (2.7, 7.5)
Nigeria	Katsina	Jul-2015	Phase 1	Yes	-	-	76776	35.3 (26.3, 44.3)*	217421	1356385	4.9 (3.4, 7.0)	3.3 (2.1, 5.3)
Nigeria	Kebbi	Jul-2010	Phase 2	Yes	-	-	9106	35.3 (26.3, 44.3)*	25787	652783	11.0 (8.8, 13.7)	0.4 (0.3, 0.5)
Nigeria	Kebbi	Dec-2010	Phase 1	No	-	-	9106	35.3 (26.3, 44.3)*	25787	652783	5.8 (4.1, 8.1)	0.7 (0.5, 1.1)
Nigeria	Kebbi	Aug-2011	Phase 1	Yes	-	-	14687	35.3 (26.3, 44.3)*	41592	669038	3.4 (2.4, 4.8)	1.8 (1.2, 2.8)
Nigeria	Kebbi	Feb-2012	Phase 1	No	-	-	19333	35.3 (26.3, 44.3)*	54749	685697	3.9 (2.7, 5.6)	2.1 (1.3, 3.3)
Nigeria	Kebbi	Sep-2012	Phase 1	Yes	-	-	19333	35.3 (26.3, 44.3)*	54749	685697	5.6 (4.3, 7.4)	1.4 (1.0, 2.1)
Nigeria	Kebbi	Jul-2013	Phase 1	Yes	-	-	17777	35.3 (26.3, 44.3)*	50342	702770	2.7 (1.8, 4.2)	2.6 (1.6, 4.6)
Nigeria	Kebbi	Mar-2014	Phase 1	No	-	-	26431	30.5 (20.6, 40.3)	86784	720269	5.9 (4.4, 8.1)	2.0 (1.4, 3.5)
Nigeria	Kebbi	Jul-2015	Phase 1	Yes	-	-	30259	35.3 (26.3, 44.3)*	85690	738204	3.3 (2.2, 4.9)	3.5 (2.2, 5.9)
Nigeria	Sokoto	Aug-2010	Phase 2	Yes	-	-	4771	35.3 (26.3, 44.3)*	13511	743238	5.3 (3.7, 7.4)	0.3 (0.2, 0.5)
Nigeria	Sokoto	Dec-2010	Phase 1	No	-	-	4771	35.3 (26.3, 44.3)*	13511	743238	5.2 (3.7, 7.4)	0.3 (0.2, 0.6)
Nigeria	Sokoto	Aug-2011	Phase 1	Yes	-	-	16261	35.3 (26.3, 44.3)*	46049	761744	4.2 (3.0, 5.9)	1.4 (0.9, 2.2)
Nigeria	Sokoto	Mar-2012	Phase 1	No	-	-	18621	35.3 (26.3, 44.3)*	52733	780712	4.4 (3.1, 6.2)	1.5 (1.0, 2.4)
Nigeria	Sokoto	Sep-2012	Phase 1	Yes	-	-	18621	35.3 (26.3, 44.3)*	52733	780712	8.8 (7.1, 10.9)	0.8 (0.6, 1.1)
Nigeria	Sokoto	Aug-2013	Phase 1	Yes	-	-	11776	35.3 (26.3, 44.3)*	33348	800151	3.6 (2.5, 5.1)	1.2 (0.7, 1.9)
Nigeria	Sokoto	Mar-2014	Phase 1	No	-	-	20493	48.3 (41.7, 55.0)	42402	820075	3.4 (2.3, 5.1)	1.5 (1.0, 2.4)
Nigeria	Sokoto	Jul-2015	Phase 1	Yes	-	-	25799	27.1 (19.5, 34.7)	95208	840495	5.3 (3.8, 7.3)	2.1 (1.4, 3.4)
Nigeria	Yobe	Aug-2010	Phase 3	Yes	-	-	2642	35.3 (26.3, 44.3)*	7482	435800	7.3 (5.4, 9.9)	0.2 (0.2, 0.4)
Nigeria	Yobe	Dec-2010	Phase 1	No	-	-	2642	35.3 (26.3, 44.3)*	7482	435800	3.2 (2.0, 5.3)	0.5 (0.3, 1.0)
Nigeria	Yobe	Aug-2011	Phase 1	Yes	-	-	11528	33.0 (23.8, 42.2)	34944	446652	1.9 (1.2, 3.0)	4.1 (2.4, 7.0)
Nigeria	Yobe	Mar-2012	Phase 1	No	-	-	23978	35.3 (26.3, 44.3)*	67903	457773	4.1 (2.9, 5.8)	3.6 (2.4, 5.6)

Country	Sub-national location	Date	IPC Phase	Prevalence estimate taken during hungry season	MAM treatment available	Acute emergency	SAM treatment admissions (n)	Coverage estimate (%); [*denotes country average]	Estimated incident cases (n)	Population (n)	Prevalence (%)	K estimate
Nigeria	Yobe	Sep-2012	Phase 2	Yes	-	-	23978	35.3 (26.3, 44.3)*	67903	457773	3.8 (2.6, 5.4)	3.9 (2.5, 6.3)
Nigeria	Yobe	Aug-2013	Phase 3	Yes	-	-	23230	34.8 (23.7, 45.8)	66786	469172	2.4 (1.5, 3.7)	6.1 (3.5, 10.9)
Nigeria	Yobe	Mar-2014	Phase 3	No	-	-	34262	39.7 (30.7, 48.7)	86370	480854	3.5 (2.3, 5.2)	5.1 (3.3, 8.1)
Nigeria	Yobe	Jul-2015	Phase 4	Yes	-	-	52394	35.3 (26.3, 44.3)*	148374	492827	3.4 (2.3, 5.2)	8.7 (5.6, 14.5)
Nigeria	Zamfara	Aug-2010	Phase 2	Yes	-	-	1135	35.3 (26.3, 44.3)*	3214	658126	4.8 (3.4, 6.8)	0.1 (0.1, 0.2)
Nigeria	Zamfara	Dec-2010	Phase 1	No	-	-	1135	35.3 (26.3, 44.3)*	3214	658126	7.4 (5.7, 9.6)	0.1 (0.0, 0.1)
Nigeria	Zamfara	Aug-2011	Phase 1	Yes	-	-	8128	35.3 (26.3, 44.3)*	23018	674513	6.1 (4.7, 8.0)	0.6 (0.4, 0.8)
Nigeria	Zamfara	Feb-2012	Phase 1	No	-	-	17005	35.3 (26.3, 44.3)*	48156	691309	4.0 (2.8, 5.6)	1.8 (1.2, 2.8)
Nigeria	Zamfara	Sep-2012	Phase 1	Yes	-	-	17005	35.3 (26.3, 44.3)*	48156	691309	5.6 (4.3, 7.4)	1.2 (0.9, 1.9)
Nigeria	Zamfara	Aug-2013	Phase 1	Yes	-	-	23907	35.3 (26.3, 44.3)*	67702	708522	4.0 (2.9, 5.7)	2.4 (1.6, 3.7)
Nigeria	Zamfara	Mar-2014	Phase 1	No	-	-	30349	39.6 (30.7, 48.5)	76718	726164	2.8 (1.8, 4.4)	3.7 (2.3, 6.3)
Nigeria	Zamfara	Jul-2015	Phase 1	Yes	-	-	33601	35.3 (26.3, 44.3)*	95154	744246	3.3 (2.2, 5.1)	3.8 (2.4, 6.1)
Somalia	Bakool Pastoral	Dec-2015	Phase 3	No	-	-	3228	55.6 (45.2, 65.9)*	5807	6920	2.2 (1.3, 3.7)	37.7 (22.0, 62.9)
Somalia	Beledweyne Rural	Jul-2016		No	-	-	1435	55.6 (45.2, 65.9)*	2582	17084	6.1 (4.4, 8.5)	2.5 (1.7, 3.7)
South Sudan	Gogrial East County	Jun-2016	Phase 3	Yes	Yes	Yes	2622	40.8 (29.6, 53.5)*	6426	27403	8.0 (5.9, 10.8)	2.9 (2.0, 4.6)
South Sudan	Ikotos County	Jan-2017	Phase 3	No	Yes	Yes	1580	40.8 (29.6, 53.5)*	3873	27393	9.1 (6.8, 12.1)	1.6 (1.0, 2.4)
South Sudan	Ikotos County	Nov-2017	Phase 3	No	Yes	Yes	1441	40.8 (29.6, 53.5)*	3532	27393	3.5 (2.3, 5.3)	3.7 (2.1, 6.0)
South Sudan	Tonj North County	Jun-2016	Phase 2	Yes	Yes	Yes	1616	40.8 (29.6, 53.5)*	3961	43948	2.0 (1.2, 3.3)	4.6 (2.5, 8.3)
South Sudan	Tonj North County	Jul-2017	Phase 3	Yes	Yes	Yes	2060	40.8 (29.6, 53.5)*	5049	45558	5.4 (4.1, 7.1)	2.1 (1.4, 3.2)
Uganda	Abim district, Karamoja Region	May-2016	Phase 2	Yes	Yes	Yes	466	34.4 (29.6, 40.7)*	1355	18299	1.5 (0.9, 2.6)	4.9 (2.7, 8.4)
Uganda	Abim district, Karamoja Region	Dec-2016	Phase 2	No	Yes	Yes	565	34.4 (29.6, 40.7)*	1642	18299	4.3 (2.6, 6.9)	2.1 (1.3, 3.5)
Uganda	Abim district, Karamoja Region	Dec-2017	Phase 2	No	Yes	Yes	564	34.4 (29.6, 40.7)*	1640	18874	2.4 (1.5, 3.6)	3.7 (2.3, 5.7)
Uganda	Abim district, Karamoja Region	Jul-2018	Phase 1	Yes	Yes	No	564	34.4 (29.6, 40.7)*	1640	19468	2.8 (1.9, 4.2)	3.0 (1.9, 4.8)
Uganda	Amudat district, Karamoja Region	May-2016	Phase 2	Yes	Yes	Yes	339	34.4 (29.6, 40.7)*	985	17926	3.3 (2.0, 5.3)	1.7 (1.0, 2.7)

Country	Sub-national location	Date	IPC Phase	Prevalence estimate taken during hungry season	MAM treatment available	Acute emergency	SAM treatment admissions (n)	Coverage estimate (%); [*denotes country average]	Estimated incident cases (n)	Population (n)	Prevalence (%)	K estimate
Uganda	Amudat district, Karamoja Region	Dec-2016	Phase 2	No	Yes	Yes	274	34.4 (29.6, 40.7)*	797	17926	7.6 (5.5, 10.5)	0.6 (0.4, 0.8)
Uganda	Amudat district, Karamoja Region	Dec-2017	Phase 2	No	Yes	Yes	233	34.4 (29.6, 40.7)*	677	18490	4.2 (3.0, 5.8)	0.9 (0.6, 1.2)
Uganda	Amudat district, Karamoja Region	Jul-2018	Phase 1	Yes	Yes	No	233	34.4 (29.6, 40.7)*	677	19072	3.7 (2.6, 5.3)	1.0 (0.6, 1.4)
Uganda	Kaabong district, Karamoja Region	May-2016	Phase 3	Yes	Yes	Yes	2078	34.4 (29.6, 40.7)*	6041	28453	4.0 (2.9, 5.6)	5.3 (3.6, 7.6)
Uganda	Kaabong district, Karamoja Region	Dec-2016	Phase 2	No	Yes	Yes	1982	34.4 (29.6, 40.7)*	5762	28453	5.1 (3.2, 8.0)	3.9 (2.5, 6.1)
Uganda	Kaabong district, Karamoja Region	Dec-2017	Phase 2	No	Yes	Yes	1153	34.4 (29.6, 40.7)*	3352	29348	4.0 (2.9, 5.5)	2.9 (2.0, 4.0)
Uganda	Kaabong district, Karamoja Region	Jul-2018	Phase 2	Yes	Yes	No	1153	34.4 (29.6, 40.7)*	3352	30272	5.9 (4.3, 8.0)	1.9 (1.3, 2.6)
Uganda	Kotido district, Karamoja Region	May-2016	Phase 2	Yes	Yes	Yes	1868	34.4 (29.6, 40.7)*	5430	30685	6.0 (4.6, 7.9)	2.9 (2.1, 3.9)
Uganda	Kotido district, Karamoja Region	Dec-2017	Phase 1	No	Yes	Yes	2056	34.4 (29.6, 40.7)*	5977	31651	4.8 (3.6, 6.5)	3.9 (2.8, 5.4)
Uganda	Kotido district, Karamoja Region	Jul-2018	Phase 2	Yes	Yes	No	2056	34.4 (29.6, 40.7)*	5977	32647	4.1 (2.8, 5.8)	4.5 (3.1, 6.6)
Uganda	Moroto district, Karamoja Region	May-2016	Phase 3	Yes	Yes	Yes	965	34.4 (29.6, 40.7)*	2805	17530	5.0 (3.5, 7.2)	3.2 (2.1, 4.9)
Uganda	Moroto district, Karamoja Region	Dec-2017	Phase 2	No	Yes	Yes	750	34.4 (29.6, 40.7)*	2180	18082	5.1 (3.7, 7.0)	2.4 (1.7, 3.4)
Uganda	Moroto district, Karamoja Region	Jul-2018	Phase 2	Yes	Yes	No	750	34.4 (29.6, 40.7)*	2180	18651	3.3 (2.2, 5.0)	3.5 (2.2, 5.7)
Uganda	Nakapiripirit district, Karamoja Region	May-2016	Phase 2	Yes	Yes	Yes	1287	34.4 (29.6, 40.7)*	3741	26556	4.7 (3.1, 6.9)	3.0 (1.9, 4.6)

Country	Sub-national location	Date	IPC Phase	Prevalence estimate taken during hungry season	MAM treatment available	Acute emergency	SAM treatment admissions (n)	Coverage estimate (%); [*denotes country average]	Estimated incident cases (n)	Population (n)	Prevalence (%)	K estimate
Uganda	Nakapiripirit district, Karamoja Region	Dec-2016	Phase 2	No	Yes	Yes	1161	34.4 (29.6, 40.7)*	3375	26556	4.7 (3.5, 6.3)	2.7 (1.9, 3.8)
Uganda	Nakapiripirit district, Karamoja Region	Dec-2017	Phase 2	No	Yes	Yes	827	34.4 (29.6, 40.7)*	2404	27392	4.4 (3.1, 6.2)	2.0 (1.3, 2.9)
Uganda	Nakapiripirit district, Karamoja Region	Jul-2018	Phase 2	Yes	Yes	No	827	34.4 (29.6, 40.7)*	2404	28254	3.5 (2.0, 5.9)	2.5 (1.4, 4.3)
Uganda	Napak district, Karamoja Region	May-2016	Phase 3	Yes	Yes	Yes	908	34.4 (29.6, 40.7)*	2640	24105	4.7 (3.3, 6.7)	2.3 (1.5, 3.5)
Uganda	Napak district, Karamoja Region	Dec-2016	Phase 2	No	Yes	Yes	853	34.4 (29.6, 40.7)*	2480	24105	7.2 (5.3, 9.8)	1.4 (1.0, 2.0)
Uganda	Napak district, Karamoja Region	Dec-2017	Phase 2	No	Yes	Yes	644	34.4 (29.6, 40.7)*	1872	24863	2.8 (1.9, 4.2)	2.7 (1.7, 4.2)
Uganda	Napak district, Karamoja Region	Jul-2018	Phase 2	Yes	Yes	No	644	34.4 (29.6, 40.7)*	1872	25646	3.8 (2.9, 4.8)	1.9 (1.4, 2.6)
Yemen	Abyan Governorate	Jan-2013	Phase 4	-	-	-	10805	61.6 (51.5, 71.7)*	17541	60838	3.2 (2.5, 4.1)	9.1 (6.6, 12.2)
Yemen	Abyan Governorate	Dec-2016	Phase 4	-	-	-	6838	61.6 (51.5, 71.7)*	11101	64192	3.1 (1.8, 5.1)	5.6 (3.3, 9.7)
Yemen	Abyan Governorate	Jan-2018	Phase 4	-	-	-	7263	61.6 (51.5, 71.7)*	11791	66530	1.8 (1.2, 3.0)	9.6 (5.9, 15.1)
Yemen	Aden Governorate	Aug-2015	Phase 4	-	-	-	5370	61.6 (51.5, 71.7)*	8718	142967	3.2 (2.4, 4.2)	1.9 (1.4, 2.7)
Yemen	Aden Governorate	Dec-2016	Phase 3	-	-	-	3400	61.6 (51.5, 71.7)*	5519	145548	5.5 (3.8, 8.0)	0.7 (0.5, 1.0)
Yemen	Al-Baidha Governorate	Oct-2015	Phase 4	-	-	-	6504	61.6 (51.5, 71.7)*	10558	110120	1.0 (0.4, 2.1)	9.9 (4.4, 25.5)
Yemen	Al-Baidha Governorate	Dec-2016	Phase 4	-	-	-	4760	61.6 (51.5, 71.7)*	7727	112107	2.4 (1.3, 4.3)	2.9 (1.6, 4.9)
Yemen	Al-Dale'e Governorate	Dec-2016	Phase 4	-	-	-	8934	61.6 (51.5, 71.7)*	14503	110766	3.3 (2.0, 5.4)	3.9 (2.3, 6.5)
Yemen	Al-Jawf Governorate	Dec-2016	Phase 3	-	-	-	3249	61.6 (51.5, 71.7)*	5274	99266	4.6 (2.9, 7.1)	1.2 (0.7, 1.9)
Yemen	Al-Mahweet Governorate	Jun-2013	Phase 3	-	-	-	3038	61.6 (51.5, 71.7)*	4932	91299	3.4 (2.6, 4.4)	1.6 (1.2, 2.2)

Country	Sub-national location	Date	IPC Phase	Prevalence estimate taken during hungry season	MAM treatment available	Acute emergency	SAM treatment admissions (n)	Coverage estimate (%); [*denotes country average]	Estimated incident cases (n)	Population (n)	Prevalence (%)	K estimate
Yemen	Al-Mahweet Governorate	Dec-2016	Phase 3	-	-	-	6655	61.6 (51.5, 71.7)*	10804	96332	4.5 (3.0, 6.7)	2.5 (1.6, 4.3)
Yemen	Amran Governorate	Sep-2013	Phase 3	-	-	-	5163	61.6 (51.5, 71.7)*	8381	162157	2.5 (1.9, 3.4)	2.1 (1.5, 3.0)
Yemen	Amran Governorate	Dec-2016	Phase 3	-	-	-	12992	61.6 (51.5, 71.7)*	21091	171098	1.9 (1.0, 3.6)	6.4 (3.2, 12.1)
Yemen	Dhamar Governorate	Mar-2013	-	-	-	-	6352	61.6 (51.5, 71.7)*	10312	327007	2.5 (1.9, 3.3)	1.2 (0.9, 1.7)
Yemen	Dhamar Governorate	Dec-2016	Phase 3	-	-	-	19937	61.6 (51.5, 71.7)*	32365	345036	4.8 (3.2, 7.0)	2.0 (1.3, 3.0)
Yemen	Hajja Governorate	May-2014	Phase 4	-	-	-	21421	61.6 (51.5, 71.7)*	34774	312047	3.0 (2.2, 4.0)	3.7 (2.7, 5.3)
Yemen	Hajja Governorate	Sep-2015	Phase 4	-	-	-	28958	61.6 (51.5, 71.7)*	47010	317680	4.3 (3.3, 5.6)	3.4 (2.5, 4.7)
Yemen	Hajja Governorate	Dec-2016	Phase 4	-	-	-	21355	61.6 (51.5, 71.7)*	34667	323414	3.5 (2.2, 5.5)	3.1 (1.9, 5.0)
Yemen	Houdeidah Governorate	Mar-2014	Phase 3	-	-	-	42361	61.6 (51.5, 71.7)	68768	502048	4.7 (3.7, 5.9)	2.9 (2.2, 3.9)
Yemen	Houdeidah Governorate	Aug-2015	Phase 4	-	-	-	62005	61.6 (51.5, 71.7)*	100657	511109	11.2 (9.0, 13.9)	1.8 (1.3, 2.4)
Yemen	Houdeidah Governorate	Dec-2016	Phase 4	-	-	-	70047	61.6 (51.5, 71.7)*	113713	520335	6.8 (4.8, 9.5)	3.2 (2.2, 4.9)
Yemen	Ibb Governorate	Dec-2016	Phase 3	-	-	-	17981	61.6 (51.5, 71.7)*	29190	396334	2.6 (1.4, 4.6)	2.8 (1.6, 5.1)
Yemen	Ibb Governorate	Mar-2017	Phase 3	-	-	-	20011	61.6 (51.5, 71.7)*	32485	403488	1.5 (0.9, 2.4)	5.5 (3.2, 9.5)
Yemen	Lahj Governorate	Jul-2014	Phase 4	-	-	-	5267	61.6 (51.5, 71.7)*	8550	124285	1.9 (1.3, 2.7)	3.7 (2.4, 5.7)
Yemen	Lahj Governorate	Oct-2015	Phase 4	-	-	-	6746	61.6 (51.5, 71.7)*	10951	126528	3.6 (2.6, 5.0)	2.4 (1.7, 3.5)
Yemen	Lahj Governorate	Dec-2016	Phase 4	-	-	-	10611	61.6 (51.5, 71.7)*	17226	128812	4.2 (2.7, 6.6)	3.2 (2.0, 5.2)
Yemen	Lahj Governorate	Jul-2017	Phase 4	-	-	-	10124	61.6 (51.5, 71.7)*	16435	131137	4.1 (3.0, 5.5)	3.1 (2.2, 4.4)
Yemen	Mareb Governorate	Dec-2016	Phase 3	-	-	-	2440	61.6 (51.5, 71.7)*	3961	47007	1.4 (0.7, 3.0)	5.8 (2.7, 12.3)
Yemen	Rayma Governorate	Dec-2016	Phase 3	-	-	-	3468	61.6 (51.5, 71.7)*	5630	90121	3.2 (1.9, 5.3)	2.0 (1.1, 3.5)
Yemen	Sa'ada Governorate	Jun-2014	Phase 4	-	-	-	9956	61.6 (51.5, 71.7)*	16162	171892	5.4 (4.4, 6.8)	1.7 (1.3, 2.3)
Yemen	Sa'ada Governorate	May-2016	Phase 4	-	-	-	18937	61.6 (51.5, 71.7)*	30742	178153	5.0 (3.9, 6.4)	3.4 (2.6, 4.7)

Country	Sub-national location	Date	IPC Phase	Prevalence estimate taken during hungry season	MAM treatment available	Acute emergency	SAM treatment admissions (n)	Coverage estimate (%); [*denotes country average]	Estimated incident cases (n)	Population (n)	Prevalence (%)	K estimate
Yemen	Sana'a Governorate	May-2016	Phase 3	-	-	-	10843	61.6 (51.5, 71.7)*	17602	236959	4.4 (3.3, 5.7)	1.7 (1.2, 2.4)
Yemen	Shabwa Governorate	Jan-2017	Phase 4	-	-	-	5558	61.6 (51.5, 71.7)*	9023	99590	1.3 (0.8, 2.1)	7.0 (4.1, 12.1)
Yemen	Taiz Governorate	May-2014	Phase 4	-	-	-	17522	61.6 (51.5, 71.7)*	28445	463186	3.1 (2.5, 3.9)	2.0 (1.5, 2.6)
Yemen	Taiz Governorate	May-2016	Phase 4	-	-	-	21180	61.6 (51.5, 71.7)*	34383	480057	5.2 (4.3, 6.4)	1.4 (1.1, 1.8)

Supplemental Table 4. Stratified analyses of incidence correction factor estimates by Integrated Food Security Phase Classification

	Phase 1-2			Phase 3-4			
**Geographic Area**	**N**	**K**	**95% CI**	**N**	**K**	**95% CI**	**p***
**All available**	230	3.3	(2.8, 3.8)	103	3.9	(3.2, 4.7)	0.085
**Western and Central Africa**	172	3.4	(2.8, 4.0)	18	5.9	(2.5, 14.2)	0.021
Burkina Faso	31	4.0	(3.4, 4.6)	0	---	---	---
Chad	20	10.9	(6.0, 19.6)	6	38.7	(22.5, 66.6)	0.027
Democratic Republic of Congo	1	6.2	(3.6, 11.6)	0	---	---	---
Liberia	15	5.9	(3.2, 11.1)	0	---	---	---
Mali	32	4.6	(3.7, 5.6)	0	---	---	---
Niger	14	7.6	(5.8, 10.0)	2	14.8	(7.8, 28.2)	0.014
Nigeria	59	1.2	(1.0, 1.6)	10	1.6	(0.7, 3.8)	0.661
**Eastern and Southern Africa**	52	3.2	(2.5, 4.0)	45	4.3	(3.4, 5.5)	0.035
Burundi	1	30.1	(7.0, 106.4)	0	---	---	---
Ethiopia	16	6.7	(4.8, 9.5)	13	6.6	(4.8, 9.1)	0.947
Kenya	9	1.5	(0.7, 3.1)	18	2.7	(1.9, 3.8)	0.036
Madagascar	2	9.3	(6.5, 13.4)	6	8.2	(6.0, 11.3)	0.664
Somalia	0	---	---	1	2.5	(1.7, 3.7)	---
South Sudan	1	3.7	(2.1, 6.0)	4	2.4	(1.7, 3.4)	0.301
Uganda	23	2.3	(1.8, 2.8)	3	3.4	(2.1, 5.5)	0.174
**Middle East and North Africa**	0	---	---	34	2.9	(2.4, 3.5)	---
Yemen	0	---	---	34	2.9	(2.4, 3.5)	---
**South Asia**	6	1.9	(0.7, 5.0)	6	2.2	(1.0, 5.3)	0.589
Afghanistan	6	1.9	(0.7, 5.0)	6	2.2	(1.0, 5.3)	0.589

*p value for Cochrane's Q statistic assessing heterogeneity between K estimates.
